# Estimating Impact Forces of Tail Club Strikes by Ankylosaurid Dinosaurs

**DOI:** 10.1371/journal.pone.0006738

**Published:** 2009-08-25

**Authors:** Victoria Megan Arbour

**Affiliations:** Department of Biological Sciences, University of Alberta, Edmonton, Alberta, Canada; Raymond M. Alf Museum of Paleontology, United States of America

## Abstract

**Background:**

It has been assumed that the unusual tail club of ankylosaurid dinosaurs was used actively as a weapon, but the biological feasibility of this behaviour has not been examined in detail. Ankylosaurid tail clubs are composed of interlocking vertebrae, which form the handle, and large terminal osteoderms, which form the knob.

**Methodology/Principal Findings:**

Computed tomographic (CT) scans of several ankylosaurid tail clubs referred to *Dyoplosaurus* and *Euoplocephalus*, combined with measurements of free caudal vertebrae, provide information used to estimate the impact force of tail clubs of various sizes. Ankylosaurid tails are modeled as a series of segments for which mass, muscle cross-sectional area, torque, and angular acceleration are calculated. Free caudal vertebrae segments had limited vertical flexibility, but the tail could have swung through approximately 100° laterally. Muscle scars on the pelvis record the presence of a large M. longissimus caudae, and ossified tendons alongside the handle represent M. spinalis. CT scans showed that knob osteoderms were predominantly cancellous, which would have lowered the rotational inertia of the tail club and made it easier to wield as a weapon.

**Conclusions/Significance:**

Large knobs could generate sufficient force to break bone during impacts, but average and small knobs could not. Tail swinging behaviour is feasible in ankylosaurids, but it remains unknown whether the tail was used for interspecific defense, intraspecific combat, or both.

## Introduction

**Figure 1:**
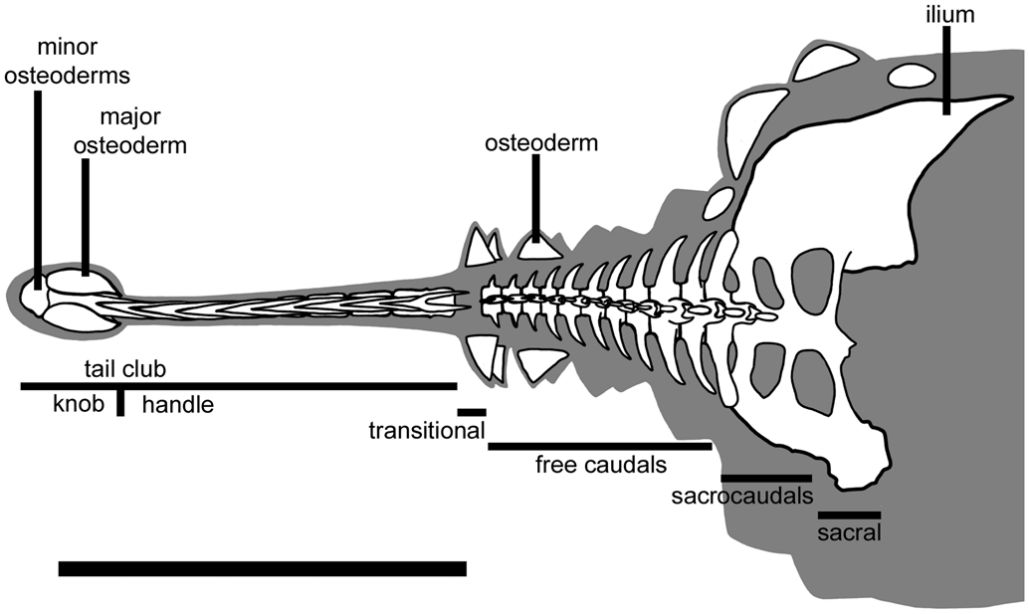
Diagram of tail terminology used in this paper. Ankylosaurid tail reconstructed from ROM 784; ROM 784 lacks the transitional caudal vertebra and the anterior portion of the pelvis. Scale bar equals 1 m. Modified from Arbour et al. (in press).

The tail club (Fig. 1) of ankylosaurid dinosaurs is composed of tightly interlocking distal caudal vertebrae, forming the handle, and large terminal osteoderms, forming the knob [1]. Parks [2] described the first ankylosaurid tail club (ROM 784, *Dyoplosaurus acutosquameus* Parks, 1924), but did not comment on its potential function. Maleev [3] interpreted the tail club of *Talarurus plicatospineus* Maleev, 1952 [3] as the ‘striking end’ of the tail, and referred to it as a mace. He later described a tail club of *Pinacosaurus grangeri* Gilmore, 1933 [4] as a double-edged axe, and suggested that the robust neural and haemal arches and presence of long ossified tendons indicated that strong muscles would have been employed in tail-swinging [5]. Coombs [6], [7], [1] discussed possible muscles associated with tail-swinging, and the possible range of motion. Thulborn [8] suggested that the tail club may have acted as a ‘dummy head’, drawing predators away from the head and neck, but this hypothesis is difficult to test. Ankylosaurids were capable of swinging the tail laterally, and the large knob and interlocking handle vertebrae suggest reinforcement against impacts. However, the biomechanics of the tail and tail-swinging in ankylosaurids have not been studied in detail.

What were the maximum force and stress that an ankylosaurid could deliver with its knob? Would the force be sufficient to damage muscle or bone in an opponent? This study examines tail club function in clubs referred to *Dyoplosaurus acutosquameus* Parks, 1924 [2], and *Euoplocephalus tutus* Lambe 1910 [9], from the Campanian of North America [10]. Functional morphology and biomechanics are examined through osteological description, computed tomography (CT) scans of several partial clubs, muscle reconstruction, and mathematical modeling of the functional dynamics of the tail.

**Institutional Abbreviations**—AMNH–American Museum of Natural History, New York, New York, USA; CMN–Canadian Museum of Nature, Gatineau, Quebec, Canada; ROM–Royal Ontario Museum, Toronto, Ontario, Canada; TMP–Royal Tyrrell Museum of Palaeontology, Drumheller, Alberta, Canada; UALVP–University of Alberta Laboratory for Vertebrate Paleontology, Edmonton, Alberta, Canada.

## Results

### Ankylosaurid Tail Osteology and Musculature

Ankylosaurid pelves are characterized by broad, horizontal ilia and a synsacrum composed of dorsosacral, sacral, and sacrocaudal vertebrae. The ilia diverge from the midline anteriorly and have a long preacetabular process and short postacetabular process. The ischia are directed ventrally or ventromedially. There are no complete pubes known for *Dyoplosaurus* or *Euoplocephalus*, but fragmentary specimens indicate that the pubis is a small, blocklike bone similar to that of nodosaurid ankylosaurs [7] and basal ankylosaurids such as *Gargoyleosaurus* (DMNH 27726).

Ankylosaurid caudal vertebrae are here divided into three categories (Fig. 1): free caudal vertebrae, which make up the anterior third to half of the tail, handle vertebrae with tightly interlocking prezygapophyses and neural spines (terminology sensu [1]), and a transitional vertebra intermediate in morphology between the two. Ankylosaurid free caudal vertebrae typically have centra that are approximately as wide as tall. In *Euoplocephalus*, centrum shape varies from circular or subcircular in anterior view; centra are subcircular in anterior view in *Dyoplosaurus*. In both taxa, neural spines are dorsoposteriorly directed, haemal spines are ventroposteriorly directed, and transverse processes are anterolaterally directed. Neural spines, haemal spines, and transverse processes are blade-like and taper distally. Neural spines, transverse processes, and postzygapophyses decrease in size posteriorly. Postzygapophyses are absent on the transitional free caudal vertebra. Transverse processes are found on all of the free caudal vertebrae.

Handle vertebrae are highly modified compared to the free caudal vertebrae (Fig. 1). The centra are anteroposteriorly elongate. Neural spines are long and low and are embraced by the elongate prezygapophyses of the successive vertebrae. Postzygapophyses are absent in the handle. Transverse processes are generally absent, but some specimens exhibit small knobs or ridges on the first few handle vertebrae that correspond to the location of the transverse processes. Ossified tendons are only found associated with the handle vertebrae and can be grouped into at least two distinct sets, which will be discussed along with the muscle reconstructions.

**Figure 2:**
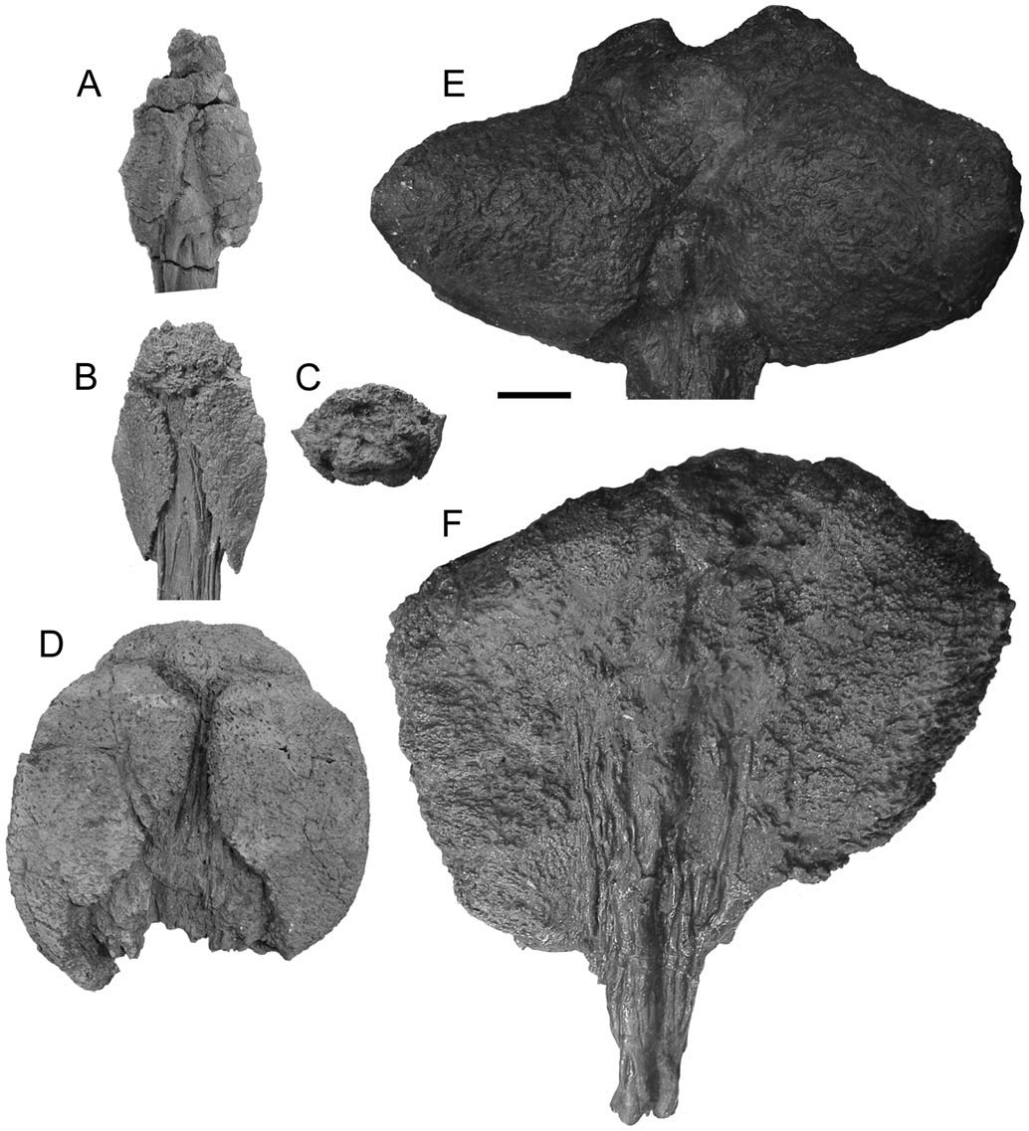
Morphology of ankylosaurid tail clubs. A) UALVP 47273, dorsal view. B) ROM 784 dorsal view and C) posterior view, D) UALVP 16247 dorsal view, E) AMNH 5245 dorsal view, and F) ROM 788 ventral view.

Tail clubs are composed of both the handle vertebrae and the large terminal osteoderms that surround and partially enclose the distalmost vertebrae, forming the knob (Fig. 1). All knobs include two major osteoderms, one on each side of the handle vertebrae, as well as a variable number of minor osteoderms that form the distal end of the knob. Knob shape is highly variable, both among and within taxa. Knobs range in width from small (<200 mm), to average (200–500 mm), to large (>500 mm) [11] (Fig. 2). Many major osteoderms have distinct longitudinal keels at the midheight or higher, with a laterally or dorsolaterally-directed axis (Fig. 2). Major osteoderms extend closer to the midline on the dorsal side than on the ventral side of the knob (Fig. 2).

### Description of Club Internal Morphology from CT Scans

CT scans provide information about the internal structure of the handle vertebrae, the knob osteoderms, and the relationships between the vertebrae, ossified tendons, and knob, as well as information about the differences between small and large clubs. UALVP 47273 (*Euoplocephalus*) provided the best data, because of the quality of the scan and because it is relatively complete. ROM 788 (*Euoplocephalus*) was scanned in two pieces (knob and handle). The knob width was only slightly smaller than the aperture of the scanner, and was slightly larger than the field of view. As a result, the lateral edges of both major osteoderms were partially excluded from the scan. Most of the knob was obscured by artifacts resulting from beam hardening and the partial volume effect [12], possibly caused by ferrous minerals infilling the pore spaces in the knob, or because the knob was too large for the X-rays to penetrate uniformly. Even with the artifacts, the borders of the specimen can usually be determined, except for the dorsal border of the vertebra in the centre of the knob. Some artifacts are present in the scan of UALVP 16247 (*Euoplocephalus*), but these are not prominent and are easily distinguished from the bone.

**Figure 3:**
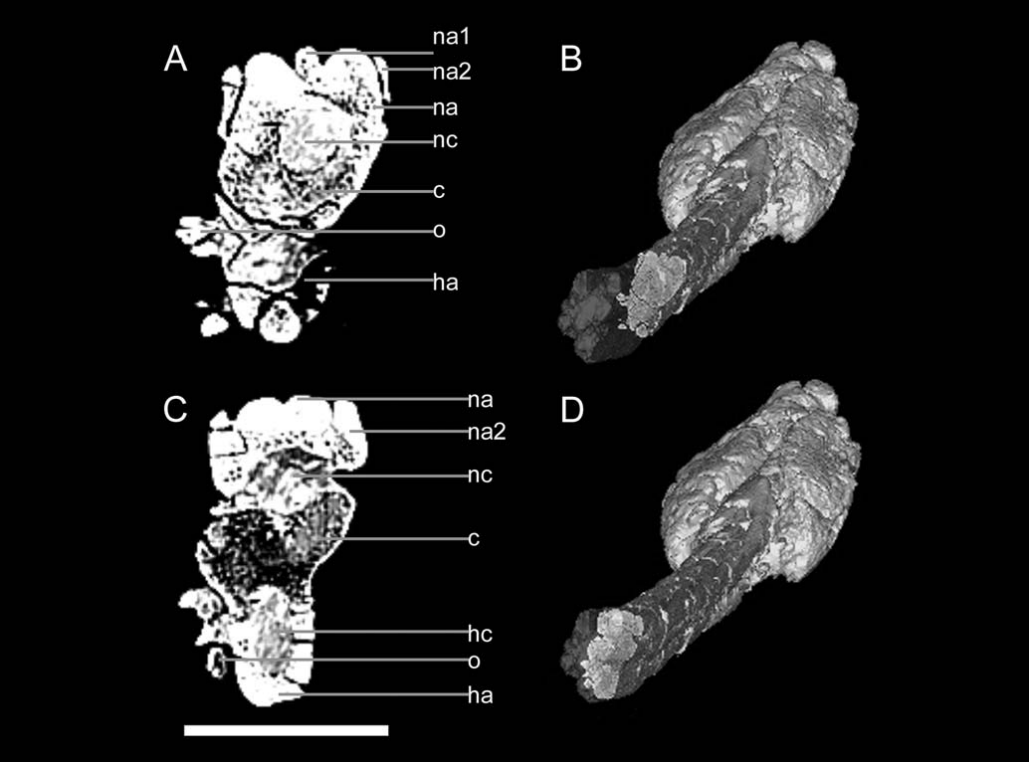
CT scan images of transverse slices through UALVP 47273 handle vertebrae in anterior view, dorsal is up. A) Midlength of a vertebra, with B) position in specimen, oblique view, anterior is to the left. C) Posterior to midlength of vertebra, with D) position in specimen. Scale bar in A and C equals 5 cm. Three-dimensional reconstructions in B and D created in Mimics. Abbreviations are as follows: c, centrum; ha, haemal arch; hc, haemal canal; na, neural arch of the centrum in the slice; na1, neural spine of the anterior vertebra; na2, prezygapophyses of the posterior vertebra; nc, neural canal; o, ossified tendon.

**Figure 4:**
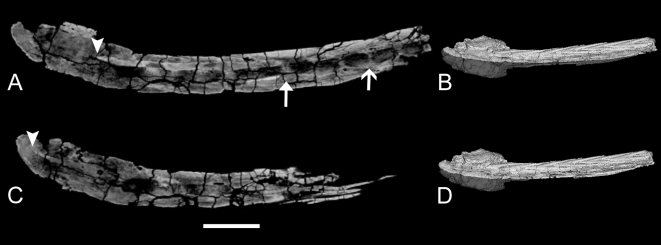
CT scan images of sagittal slices through UALVP 47273 handle in left lateral view, dorsal is up. A) Mid-width of the club. Most of the centra appear to be fused at the anterior and proximal faces (arrow with open head), although one joint does not appear fused (arrow with closed head), with B) position in specimen, oblique dorsal view, anterior is to the right. C) Mid-width of the left half of the club, with D) position in specimen. The neural canal extends to the anterior terminus of the minor plates at the distal end of the knob (arrow). The three narrow, vertically stacked structures at the anterior of the handle are ossified tendons. Scale bar equals 10 cm. Three-dimensional reconstructions in B and D created in Mimics.

In UALVP 47273 and ROM 788, the centra are comprised of low density cancellous bone, whereas the neural and haemal arches are dense compact bone (Fig. 3). The ossified tendons are similarly dense. The neural and haemal canals are radiolucent in the scans, indicating that they have been infilled with minerals. Transversely, the neural canal is circular to oval. The haemal canal is always completely enclosed by bone. The centra are at times difficult to discern in the knob, but the neural and haemal canals are visible until near the terminus of the knob. In UALVP 47273, the neural canal seems to end at approximately the anterior border of the minor plates that comprise the distal end of the knob (Fig. 4).

**Figure 5:**
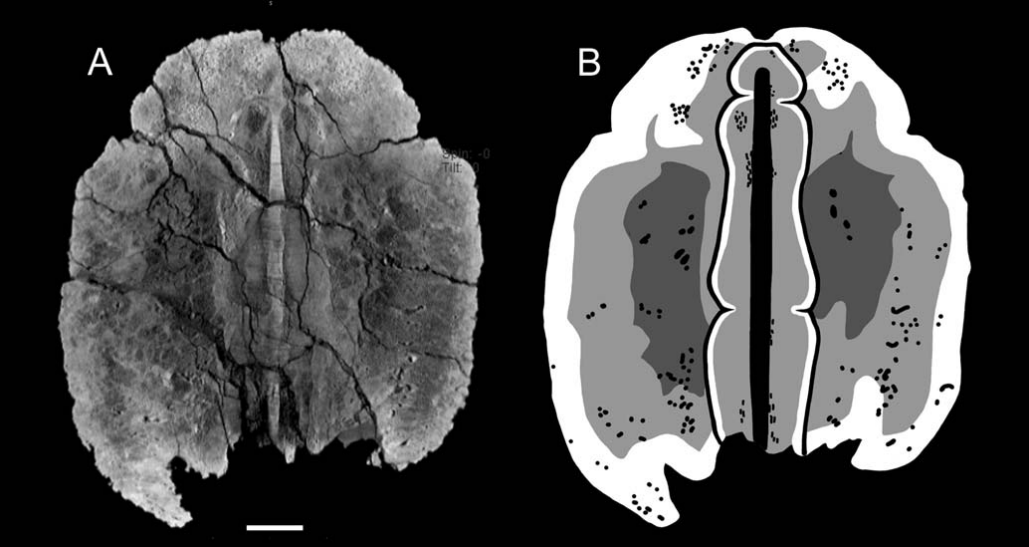
Internal anatomy of a tail club knob. A) CT scan image of coronal slices through UALVP 16247 in dorsal view, at knob mid-height, posterior is up. B) Interpretive illustration of (A), showing the shapes of the vertebrae, highest density areas (white), medium density areas (light grey), and lowest density areas (dark grey). The neural canal and vascular canals in the osteoderms are indicated by black. Scale bar equals 5 cm.

In UALVP 16247, the shape and number of the vertebrae in the knob is best viewed in coronal view (Fig. 5). Three vertebrae are preserved in the knob, and the last vertebra extends almost to the posterior terminus of the knob. The posterior two vertebrae are completely enclosed laterally by the major osteoderms. The anterior two vertebrae are partially exposed dorsally, but the terminal vertebra is dorsally covered by the minor plates. In dorsal view, the two anterior vertebrae have the characteristic elongate hourglass shape found in handle vertebrae. The terminal vertebra is abbreviated in length, with a length of less than one third that of the penultimate vertebra. The terminal vertebra is roughly triangular in dorsal view, rather than hourglass-shaped.

In some tail clubs (e.g. AMNH 5245, *Euoplocephalus*), successive tail club centra are not fused at the anterior and posterior ends. However, in sagittal view of UALVP 47273, bright zones at the articular ends of the centra, and a lack of distinct spaces between the centra, seem to indicate fusion of successive handle vertebrae (Fig. 4). Alternately, this may result from mineralization of the space between vertebrae. Vertebrae appear fused in ROM 788, although the specimen has been partially reconstructed and painted. The CT scan of the ROM 788 handle does not clarify whether or not the vertebrae are fused at the centra.

**Figure 6:**
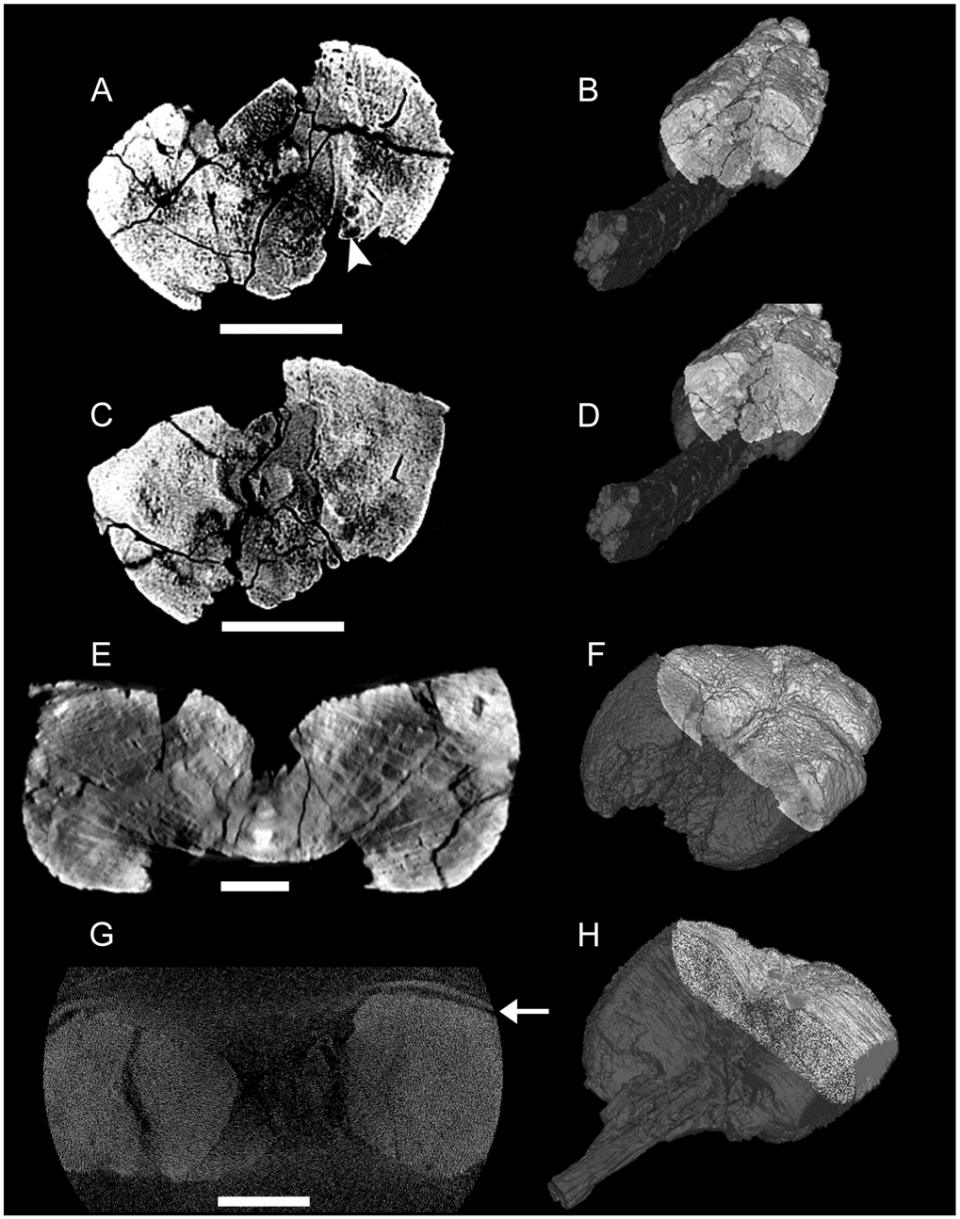
CT scan images of transverse slices through knobs, dorsal is up. A) UALVP 47273, with B) position in specimen. The arrowhead marks three vertically stacked ossified tendons between the left major osteoderm and the vertebra. C) UALVP 47273, with D) position in specimen. E) UALVP 16247, with F) position in specimen. G) ROM 788, with H) position in specimen; artifacts obscure most fine details. The arrowhead marks the CT scanning tray. Scale bars in A, C, and E equal 5 cm, scale bar in G equals 10 cm. Three-dimensional reconstructions in B, D, F, and G created in Mimics (not to scale).

Ossified tendons are preserved alongside the handles in all CT scanned specimens. In UALVP 16247 and UALVP 47273, the ossified tendons are visible between the osteoderms and vertebra (Fig. 6). Tendons along handle appear bright, but tendons enclosed by the knob osteoderms are dark.

In UALVP 47273, the osteoderms each have a relatively thin compact cortex, and are predominantly cancellous (Fig. 6). The cortex is slightly thicker on the right major plate than on the left, especially at the keel. This compact bone is absent on the dorsal and ventral medial edges of the major plates. The minor plates at the distal tip of the knob are somewhat denser than the major plates. Neurovascular channels are approximately radially oriented near the outer edges of the osteoderms, and have a more random distribution medially. Some large pores can be traced several centimeters dorsally from the ventral border of the knob osteoderms. In transverse sections through the major plates, there are patches of low density (Fig. 6). These change shape anteroposteriorly, but remain symmetrical between the osteoderms.

### Muscle Reconstructions

The intrinsic vertebral muscles Mm. interspinales and Mm. interarcuales, which connect the anterior and posterior edges of successive neural spines, and Mm. interarticulares superiores, which connect the zygapophyses of successive vertebrae, are present in both crocodilians and birds [13], and were probably present in ankylosaurids as well. M. multifidus may or may not have been present in ankylosaurids, because its presence in other ornithischians is neither supported nor refuted [13].

**Figure 7:**
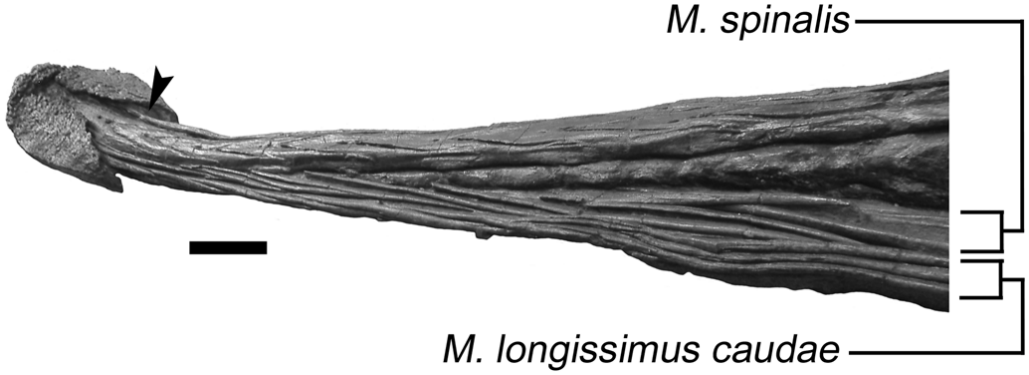
Ossified tendons in ROM 784, oblique right lateral view. M. spinalis is represented by the inner set of imbricated tendons, and M. longissimus caudae is represented by the outer set of parallel to braided tendons. The ossified tendons continue underneath the knob osteoderms (arrowhead). Scale bar equals 10 cm.

Ossified tendons are useful for interpreting the presence of muscles in fossil skeletons because they represent part of this soft tissue complex [13], and their presence in ankylosaurid tail clubs can be used to infer the presence of some caudal musculature. Ossified tendons are known from all ankylosaurid taxa with preserved tail clubs, but are best preserved in ROM 784, *Dyoplosaurus* (Fig. 7). Ossified tendon arrangement is similar across ankylosaurid taxa, and that muscles of the tail of *Dyoplosaurus* and *Euoplocephalus* were probably similar. Parks [2] recognized three series of tendons on the dorsolateral sides of the handle, and four on the distal, ventral side of the tail. Observation of the specimen indicates that the tendons are more readily grouped into two sets on the dorsolateral sides. The ventral side of the specimen is not exposed. The inner set of tendons has an imbricated appearance, whereas the tendons of the outer layer are parallel with a braided appearance. The inner tendons are shorter in length compared to the long outer tendons, and have smaller diameters. The inner set of tendons is slightly dorsal to the outer set. Posteriorly, the inner and outer sets converge towards the knob, whereas anteriorly the two sets are distinctly separated. The tendons are posterodorsally oriented, and the inner set more strongly so. The anteriormost outer tendons are parallel and vertically stacked. The inner set of tendons inserts at either the midpoint of the centrum or the neural arch.

Coombs [1] briefly discussed ossified tendons in ankylosaurids, and suggested that caudal ossified tendons represented M. iliocaudalis, M. caudofemoralis, and various intrinsic axial muscles. Based on comparisons with the work of Organ [13] and Holmes and Organ [14], the dorsoposteriorly-oriented, inner set of ossified tendons alongside the handle probably represents M. spinalis. Organ [13] considered parallel bundles of tendons along the transverse processes as representing M. longissimus dorsi or M. iliocostalis. Because M. iliocostalis is not present along the caudal vertebrae, it is likely that the parallel, outer set of tendons in ROM 784 represents M. longissimus caudae. M. transversospinalis was present, and is represented in the distal portion of the tail by ossified tendons from the M. spinalis subunit. It is unknown whether M. semispinalis was present, and if so, how large it was in the caudal region.

**Figure 8:**
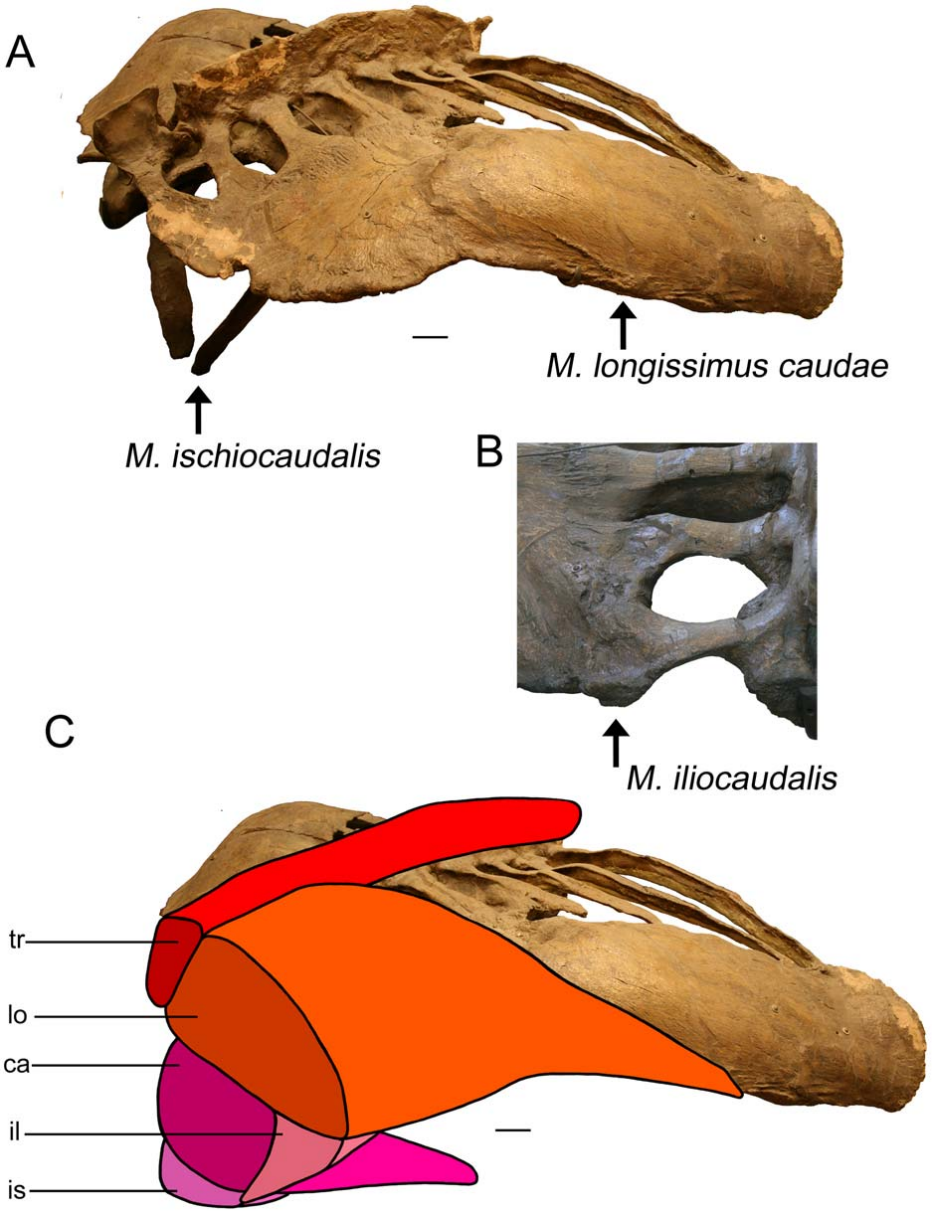
Origins of tail muscles on the pelvis. A) AMNH 5409 (*Euoplocephalus*) pelvis, posterior right dorsolateral view. M. ischiocaudalis originates at the distal terminus of the ischium. The origin of M. longissimus caudae is marked by a long, pronounced ridge and rugose area on the lateral aspect of the ilium. The posterior terminus of the ilium is partially reconstructed. B) AMNH 5337 (*Euoplocephalus*) pelvis, dorsal view, anterior up, showing the posterior terminus of the left ilium. M. iliocaudalis originates from a large knob. C) AMNH 5409, same view as (A), with reconstructed musculature. The muscles are cut posteriorly to show their relationships in cross-section. M. caudofemoralis longus originates on the transverse processes of the free caudal vertebrae, and inserts on the fourth trochanter of the femur (not shown). M. transversospinalis originates and inserts on the neural spines. Scale bars equal 10 cm. Abbreviations are as follows: ca = M. caudofemoralis longus, il = M. iliocaudalis, is = M. ischiocaudalis, lo = M. longissimus caudae, tr = M. transversospinalis.

Symmetrical ridges located approximately halfway along the lateral edge of the ilium of AMNH 5409 (Fig. 8) likely correspond to the origin of M. longissimus caudae, based on comparisons with extant crocodilians [15]. These ridges are more >5 cm long, and suggest that M. longissimus caudae was large, at least proximally. Coombs [7] suggested that the rugose lateral edges of ankylosaurid ilia corresponded to the origin of M. longissimus dorsi, although this would have resulted in an unusually long M. longissimus dorsi. The transverse processes are not large or robust in ankylosaurids, and these would have limited the size of M. longissimus caudae posteriorly along the tail. The handle vertebrae lack transverse processes, although there are occasionally bumps or ridges along the lateral sides of the centra (e.g. ROM 784), which may represent the insertion of M. longissimus caudae.

Coombs [7] reconstructed ankylosaur pelvic muscles with separate M. iliocaudalis and M. ischiocaudalis. According to Coombs [7], M. iliocaudalis originated from a massive blunt knob at the caudal end of the ilium and inserted only along the proximal caudals, and this interpretation is accepted here (Fig. 8). M. ischiocaudalis originated from the distal terminus of the ischium, and Coombs [7] suggested that this muscle was probably not involved in tail swinging, due to the vertical orientation of the ischium (Fig. 8). M. caudofemoralis longus (Fig. 8) inserted onto the distally located fourth trochanter of ankylosaurids [7].

**Figure 9:**
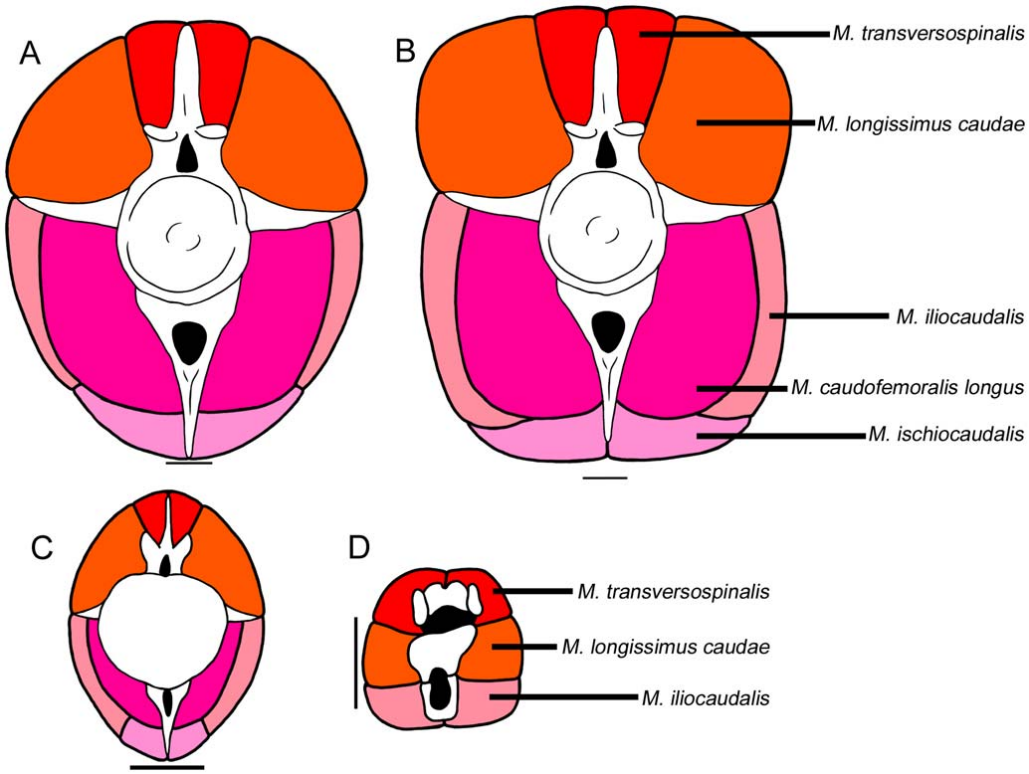
Cross-sectional reconstructions of ankylosaurid caudal musculature. A) Anterior free caudal vertebra, modified from TMP 85.26.70 (*Euoplocephalus*). M. transversospinalis is not divided into its subunits. The relative sizes of all muscles are speculative, especially M. iliocaudalis and M. ischiocaudalis. B) More muscular reconstruction, with muscles bulging past neural spine, haemal spine, and transverse processes. This reconstruction is 43% larger than the reconstruction in A. C) Posterior free caudal vertebra, reconstructed from TMP 2007.20.100. M. iliocaudalis may not have extended very far posteriorly along the tail, in which case M. ischiocaudalis may have occupied the area reconstructed as M. ischiocaudalis here. D) Musculature of the handle, reconstructed from a CT scan image of UALVP 47273 at the midlength of the club. M. transversospinalis and M. longissimus caudae are represented by ossified tendons in many tail club specimens. The size of M. iliocaudalis is speculative. The width of M. longissimus caudae is equivalent to the maximum space between the major osteoderms of the knob. Scale equals 5 cm.

Ankylosaurid tail musculature is reconstructed in cross section in figure 9 based on crocodilian tail anatomy as described in the literature. Because the morphology of various subunits of M. transversospinalis is uncertain, and because there is little osteological evidence for the size of these divisions, the entire M. transversospinalis system is depicted rather than its components. In the free caudal vertebrae, M. transversospinalis would have occupied the area closest to the neural spine. M. longissimus caudae is here reconstructed as a large muscle occupying the area lateral to M. transversospinalis to the distal terminus of the transverse process.

Ventrally, M. caudofemoralis longus is the largest muscle (Fig. 9). It is reconstructed here occupying an area between the transverse process and the stout portion of the haemal arch. Cong et al. [15] shows M. ilioischiocaudalis of *Alligator sinensis* forming the outer boundary of the ventral tail musculature, between the transverse process and the distal portion of the haemal spine. Ankylosaurids likely had a small M. ischiocaudalis [7], which is here reconstructed occupying the area near the ventral terminus of the haemal spine. Cong et al. [15] also shows that there is a varying amount of fat between the M. caudofemoralis longus and M. ilioischiocaudalis in the anterior portion of the tail, which reduces in size posteriorly. These fat deposits leave no correlates for reconstruction in ankylosaurids, and so are excluded here. In crocodilians, the musculature of the tail is to a certain extent limited by the vertebrae, but the cross-sectional profile of the tail changes greatly from anterior to posterior [15]. A conservative reconstruction of the muscles of the tail of ankylosaurids would have an elliptical cross-sectional outline, with none of the muscles bulging past the transverse processes, or neural and haemal spines.

There are fewer osteological correlates for muscle attachments in the handle vertebrae, and it is even more difficult to estimate the cross-sectional outline of the muscles than in the free caudal vertebrae. However, one clue that may indicate muscle area is the amount of space between the knob osteoderms. Their bumpy or dendritic texture suggests that they were covered by a keratinous sheath, and not muscle. In crocodilians, the epaxial musculature is firmly connected to the dermis [16], and tendons of M. spinalis insert on the basal sides of osteoderms [13]. The width between the two major knob osteoderms in dorsal view must have been the maximum width of the handle muscles. M. transversospinalis is represented by ossified tendons in the handle, and probably occupied the area dorsal and lateral to the neural arch. The outer set of ossified tendons in ROM 784 may represent M. longissimus caudae, which would have occupied the space lateral to the centrum. In crocodylians and lizards M. caudofemoralis longus originates on the transverse processes of the anterior caudal vertebrae, and inserts tendinously on the fourth trochanter of the femur and to the shank [17], [18]. As such, M. caudofemoralis longus was likely absent along the handle vertebrae, because these lack transverse processes. It is unknown whether or not M. ischiocaudalis was present in this region. In these reconstructions, M. iliocaudalis occupies the space ventral and lateral to the haemal arch.

All of the epaxial musculature would function to bend the tail laterally, and an anteriorly large M. longissimus caudae might imply that the tail could be swung quite forcefully. A problem with trying to understand which muscles may have contributed the most to tail-swinging actions is the lack of understanding of tail muscle function in extant analogues. Further research on the function of large muscles in crocodilian tails would help clarify the reconstructed musculature of ankylosaurid tails.

### Estimated Impact Forces

#### ROM 784/UALVP 47273 (Dyoplosaurus/Euoplocephalus)

Estimates of volume, mass, torque, and rotational inertia are found in Tables 1–4 and are based on methods by Carpenter et al. [19], described in the materials and methods section. Using these estimates, the angular rate of movement of the ROM 784/UALVP 47273 club (ωclub) was between 4.75 rad/s and 9.38 rad/s. The length of the tail from the anterior end of the first free caudal vertebra, to the posterior end of the knob, is 216 cm. If a tail club was swung laterally, the impact site would not be at the posterior end of the knob, but somewhere along the lateral edge of one of the major plates of the knob. These osteoderms are sharply keeled laterally, and the maximum width is at approximately 16 cm from the posterior tip of the knob. Using this as an impact site, the impact site is 201 cm from the anterior face of the first free caudal. With this, the impact velocity of the club can be calculated:
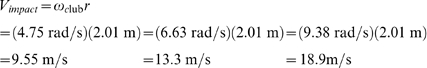
(1)


**Table 1:** Summary of volumes, areas, and masses for the ROM 784/UALVP 47273 composite tail. Muscle and bone mass are after Carpenter et al. (2005).

Segment	Muscle Cross-Sectional Area (cm^2^)	Muscle Volume (cm^3^)	Muscle Mass (g) (ρ = 1.0 g/cm^3^)	Bone Volume (cm^3^)	Bone Mass (g) (ρ = 1.98 g/cm^3^)	Total Mass (g)	Length (cm)	Mass per unit length (g/cm)
**1**	2526	18070	18070	1514	2998	21070	7.59	2776
**2**	2239	15880	15880	1447	2864	18740	7.56	2479
**3**	1965	13810	13810	1381	2734	16550	7.53	2197
**4**	1706	11860	11860	1317	2607	14470	7.50	1929
**5**	1460	10030	10030	1255	2484	12520	7.47	1675
**6**	1228	8321	8321	1194	2364	10680	7.44	1435
**7**	1011	6724	6724	1135	2247	8972	7.42	1210
**8**	807	5243	5243	1078	2134	7377	7.39	999
**9**	617	3874	3874	1022	2024	5898	7.36	802
**10**	441	2699	2699	968.2	1917	4616	7.33	630
**11**	300	2089	2089	915.9	1813	3902	7.30	535
**Club**	273	9357	9357	5346	10580	19940	134	148

Segment numbers refer to each free caudal vertebra and associated muscle, with the final club segment composed of the handle vertebrae, knob, and associated muscle.

**Table 2 pone-0006738-t002:** Rotational inertias for each segment of the ROM 784/UALVP 47273 composite tail.

Seg-ment	I_tail_	I_tail-1_	I_tail-1-2_	I_tail-1-2-3_	I_tail-1-2-3-4_	I_tail-1-2-3-4-5_	I_tail-1-2-3-4-5-6_	I_tail-1-2-3-4-5-6-7_	I_tail-1-2-3-4-5-6-7-8_	I_tail-1-2-3-4-5-6-7-8-9_	I_tail-1-2-3-4-5-6-7-8-9-10_	I_tail-1-2-3-4-5-6-7-8-9-10-11_
**1**	4.043e5	n/a										
**2**	2.511e6	3.570e5										
**3**	5.996e6	2.200e6	3.127e5									
**4**	1.017e7	5.204e6	1.909e6	2.714e5								
**5**	1.445e7	8.734e6	4.467e6	1.639e6	2.329e5							
**6**	1.834e7	1.225e6	7.399e6	3.784e6	1.388e6	1.973e5						
**7**	2.141e7	1.528e7	1.020e7	6.164e6	3.153e6	1.157e6	1.644e5					
**8**	2.333e7	1.747e7	1.247e7	8.324e6	5.029e6	2.572e6	9.437e5	1.341e5				
**9**	2.387e7	1.851e7	1.386e7	9.892e6	6.605e6	3.990e6	2.041e6	7.487e5	1.064e5	n/a		
**10**	2.324e7	1.853e7	1.438e7	1.076e7	7.682e6	5.129e6	3.099e6	1.585e6	5.813e5	8.2630e4		
**11**	2.390e7	1.949e7	1.555e7	1.206e7	9.028e6	6.443e6	4.302e6	2.599e6	1.329e6	4.876e5	6.931e4	
**Club**	4.735e8	4.295e8	3.880e8	3.489e8	3.121e8	2.778e8	2.458e8	2.162e8	1.888e8	1.637e8	1.408e8	1.202e8
**Total (g/cm^2^)**	6.411e8	5.475e8	4.685e8	4.018e8	3.453e8	2.973e8	2.564e8	2.212e8	1.908e8	1.643e8	1.409e8	1.202e8
**Total (kg/m^2^)**	6411	5475	4685	4017	3453	2973	2564	2212	1908	1643	1409	1202

Segment numbers refer to each free caudal vertebra and associated muscle, with the final club segment composed of the handle vertebrae, knob, and associated muscle.

**Table 3 pone-0006738-t003:** Muscle cross-sectional areas, muscle forces, and torques for each segment of the ROM 784/UALVP 47273 composite tail.

Segment	Muscle cross-sectional area at proximal end of segment (cm^2^)	Force (half of muscle cross-sectional area multiplied by 20 N/cm^2^)	Force (half of muscle cross-section multiplied by 39 N/cm^2^)	Force (half of muscle cross-section multiplied by 78 N/cm^2^)	Link half width (m)	Torque at base of link, 20 N/cm^2^ (Nm)	Torque at base of link, 39 N (Nm)	Torque at base of link, 78N (Nm)
**1**	2526	25260	49260	98520	0.1476	3728	7269	14540
**2**	2239	22390	43650	87310	0.1361	3047	5942	11880
**3**	1965	19650	38320	76640	0.1247	2450	4777	9555
**4**	1706	17060	33260	66520	0.1132	1931	3765	7530
**5**	1460	14600	28470	56940	0.1017	1485	2897	5793
**6**	1228	12280	23950	47910	0.0903	1109	2162	4325
**7**	1011	10110	19710	39410	0.0788	797	1553	3106
**8**	807	8067	15730	31460	0.0674	543	1060	2119
**9**	617	6167	12030	24050	0.0559	345	672	1344
**10**	441	4407	8593	17190	0.0444	195	382	763
**11**	300	2995	5841	11680	0.0347	104	203	405
**Club**	273	2731	5325	10650	0.0337	92	179	359

Segment numbers refer to each free caudal vertebra and associated muscle, with the final club segment composed of the handle vertebrae, knob, and associated muscle.

**Table 4:** Cumulative moment of inertias and segment angular rate of movement.

Segment	Cumulative moment of inertia	ω (rad/s) (20N)	ω (rad/s) (39N)	ω (rad/s) (78N)
**1+2+3+4+5+6+7+8+9+10+11+club**	6411	0.55	0.77	1.09
**2+3+4+5+6+7+8+9+10+11+club**	5475	0.54	0.76	1.07
**3+4+5+6+7+8+9+10+11+club**	4685	0.52	0.73	1.03
**4+5+6+7+8+9+10+11+club**	4017	0.50	0.70	0.99
**5+6+7+8+9+10+11+club**	3453	0.47	0.67	0.94
**6+7+8+9+10+11+club**	2973	0.44	0.62	0.88
**7+8+9+10+11+club**	2564	0.40	0.57	0.80
**8+9+10+11+club**	2212	0.36	0.50	0.71
**9+10+11+club**	1908	0.31	0.43	0.61
**10+11+club**	1643	0.25	0.35	0.50
**11+club**	1409	0.20	0.28	0.39
**Club**	1202	0.20	0.28	0.40
**Total**		4.75	6.63	9.38

Segment numbers refer to each free caudal vertebra and associated muscle, with the final club segment composed of the handle vertebrae, knob, and associated muscle. The angle of articulation is 15 degrees, or 0.2618 radians.

These three calculations represent the three angular momentum results using different muscle specific tension estimates. Using the mass of the club segment (19.94 kg, Table 1), the impulse delivered by the club can be calculated:
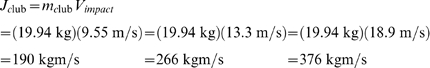
(2)


Carpenter et al. [19] assumed a stopping time of 1/3 s. Snively and Cox [20] estimated stopping times for *Pachycephalosaurus* head-butting impacts in the range of 0.05 to 0.1 s. In the case of *Pachycephalosaurus*, the stopping time could be estimated based on the mass and velocity of the impacting and impacted bodies (in this case, both *Pachycephalosaurus*). Stopping time cannot be estimated as easily because tail clubs could be used to impact a variety of objects of different masses and velocities. Therefore, a stopping time of 0.333 s is a reasonable estimate. Using this, the maximum force exerted on the target can be calculated, as the impulse/interval.
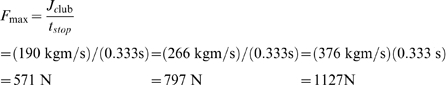
(3)


The stress exerted by the impacting club is F_max_ over the area of impact. The site of impact is the lateral keel of one of the major knob osteoderms. The amount of area involved in the impact can vary. If the sharpest part of the keel is the site of impact (height = ∼0.20 cm), and 1 cm of length is involved, then the area of impact is 0.20 cm^2^.
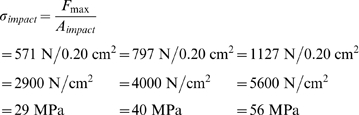
(4)


Sensitivity analyses (Tables 5–7) examined the effects of changing variables such as mass, impact area, and flexibility of the tail. Reducing the mass of the tail club segment by 15% reduced the rotational inertia of this segment, and therefore increased the angular rate of movement and impact velocity by 8%. However, the reduction in tail club segment mass also reduced both the impact force and stress by 9%. Increasing the cross-sectional area of each segment by 43% increased the mass of all segments, and increased the cross-sectional area of muscle in each segment, which in turn increased the torque of each segment. Impact force and stress each increased by 27%.

**Table 5:** Summary of results of sensitivity analyses for ROM 784/UALVP 47273– angular accelerations, velocities, and impulses.

	ω low (rad/s)	ω med (rad/s)	ω high (rad/s)	V low (m/s)	V med (m/s)	V high (m/s)	J low (kgm/s)	J med (kgm/s)	J high (kgm/s)
**Baseline**	4.75	6.63	9.38	9.54	13.3	18.8	190	266	376
**Mass**									
*Lighter*	5.12	7.15	10.12	13.4	14.4	20.3	225	241	341
**Muscles**									
*Larger*	5.08	7.09	10.0	10.2	14.3	20.2	242	337	477
**Articulation**									
*5°*	2.74	3.83	5.41	5.51	7.70	10.9	110	153	217
*10°*	3.88	5.41	7.66	7.79	10.9	15.4	155	217	307
*20°*	5.48	7.66	10.8	11.0	15.4	21.8	220	307	434
*15°-0°*	3.66	5.11	7.23	7.36	10.3	14.5	147	205	290
**Free caudals**									
*No handle*	5.95	8.31	11.75	11.9	16.7	23.6	236	330	467
**Impact site (m)**									
*1.87*	4.75	6.63	9.38	8.88	12.4	17.5	177	247	350
*2.06*	4.75	6.63	9.38	9.78	13.7	19.3	195	272	385
**Impact area (cm)**									
*0.1*	4.75	6.63	9.38	9.54	13.3	18.8	190	266	376
*0.75*	4.75	6.63	9.38	9.54	13.3	18.8	190	266	376
*2*	4.75	6.63	9.38	9.54	13.3	18.8	190	266	376
**Stopping time (s)**									
*0.033*	4.75	6.63	9.38	9.54	13.3	18.8	190	266	376
*0.1*	4.75	6.63	9.38	9.54	13.3	18.8	190	266	376

**Table 6 pone-0006738-t006:** Summary of results of sensitivity analyses for ROM 784/UALVP 47273– forces and stresses.

	F low (N)	F med (N)	F high (N)	σ low (N/cm^2^)	σ med (N/cm^2^)	σ high (N/cm^2^)
**Baseline**	571	797	1127	2900	4000	5600
**Mass**						
Lighter	675	725	1030	3370	3630	5130
**Muscles**						
Larger	726	1.01e3	1430	3629	5067	7167
**Articulation**						
5°	330	461	652	1650	2300	3260
10°	467	652	922	2330	3260	4610
20°	660	922	1300	3300	4610	6520
15°-0°	441	616	871	2200	3080	4350
**Free caudals**						
No handle	710	991	1400	356	4960	7010
**Impact site (m)**						
1.87	532	743	1050	2660	3710	5250
2.06	586	818	1160	2930	4090	5780
**Impact area (cm)**						
0.1	572	798	1130	5720	7980	11300
0.75	572	798	1130	762	1060	1500
2	572	798	1130	286	399	564
**Stopping time (s)**						
0.033	5.72e3	7.98e3	11300	28600	39900	56400
0.1	1.90e3	2.66e3	3760	9520	13300	18800

**Table 7:** Percentage difference between the baseline analyses and each sensitivity analysis for angular acceleration, impact velocity, impulse, impact force, and impact stress.

	ω (rad/s)	V (m/s)	J (kgm/s)	F (N)	σ (N/cm^2^)
**Mass**					
*Lighter*	8	8	−9	−9	−9
**Muscles**					
*Larger*	7	7	27	27	27
**Articulation**					
*5°*	−42	−42	−42	−42	−42
*10°*	−18	−18	−18	−18	−18
*20°*	15	15	15	15	15
*15°-0°*	−23	−23	−23	−23	−23
**Free caudals**					
*No handle*	25	25	25	25	25
**Impact site (m)**					
*1.87*	0	−7	−7	−7	−7
*2.06*	0	3	3	3	3
**Impact area (cm)**					
*0.1*	0	0	0	0	99
*0.75*	0	0	0	0	−73
*2*	0	0	0	0	−90
**Stopping time (s)**					
*0.033*	0	0	0	901	901
*0.1*	0	0	0	232	232

Reducing the angle of articulation reduced the angular acceleration, which resulted in lower impact velocities, impulses, impact forces, and impact stresses, whereas increasing the angle of articulation increases these variables. Decreasing the angle of articulation posteriorly along the tail also reduces these variables. The absence of a handle increased angular acceleration, impact velocity, impulse, and maximum force, and ultimately increased impact stress by 25%. These increases would likely be even greater if a more accurate tail with lengthening distal caudals could be reconstructed.

Moving the impact site anteriorly by 7% decreased the impact velocity, impulse, and maximum force, and decreased impact stress by 7%. Moving the impact site posteriorly by 2.5% increased impact stress by 3%. The area of impact only affected the impact stress, because impact stress is F_max_/A_impact_. The larger the area of impact, the greater the area over which the force is distributed, and the lower the impact stress. Reducing the area of impact from 0.20 cm^2^ to 0.10 cm^2^ increased the impact stress by 99%. An impact area of 2 cm^2^ decreased the impact stress by 90%. Altering the stopping time did not affect the impact velocity or impulse, but did affect maximum force (because this represents impulse over the stopping time) and stress. Decreasing the stopping time from a third to a tenth of a second increased impact force and stress each by 232%.

#### AMNH 5245/ROM 788 (Euoplocephalus)

The angular rate of movement of the AMNH 5245/ROM 788 club (ω_club_) is between 4.7569 rad/s and 9.3942 rad/s. The length of the tail from the anterior end of the first free caudal vertebra, to the posterior end of the knob, is 348.66 cm. If the impact site is located at approximately the maximum width of the tail (located roughly 20 cm anterior to the posterior terminus of the knob), then the impact site is 328.66 cm from the anterior face of the first free caudal vertebra. This is used to calculate the impact velocity (Eq. 1). The mass of the club segment (154.97 kg) is used to calculate the impulse delivered by the club (Eq. 2). The maximum force (Eq. 3) is calculated using a stopping time of 1/3 s, and the impact stress (Eq. 4) is calculated assuming an impact area of 0.20 cm^2^ as for ROM784/UALVP 47273. The results are summarized in Table 8.

**Table 8:** Impact velocities, impulses, forces, and stresses for the AMNH 5245/ROM 788 composite tail.

	ω_club_ = 4.76	ω_club_ = 6.64	ω_club_ = 9.39
**Velocity (m/s)**	15.66	21.85	30.89
**Impulse (kgm/s)**	2427	3387	4788
**Force (N)**	7281	10160	14360
**Stress (N/cm^2^)**	36400	50800	71810
**Stress (MPa)**	364	508	718

#### UALVP 16247 (Euoplocephalus)

The same method for calculating velocity, impulse, force and stress (Table 9) use the same equations (Eqs. 1–4) as for ROM 784/UALVP 47273, and AMNH 5245/ROM 788.

**Table 9:** Impact velocities, impulses, forces, and stresses for the UALVP 16247 reconstructed tail.

	ω_club_ = 4.76	ω_club_ = 6.64	ω_club_ = 9.39
**Velocity (m/s)**	9.45	13.20	18.67
**Impulse (kgm/s)**	321	475	671
**Force (N)**	962	1424	2014
**Stress (N/cm^2^)**	4811	7119	10070
**Stress (MPa)**	48	71	101

## Discussion

The gross and internal morphology of ankylosaurid tail clubs suggests that these structures evolved for delivering forceful impacts. Muscle scars on the pelvis suggest that a large M. longissimus caudae was present, which may have resulted in a powerful swing. Ankylosaurid caudal vertebrae are lightly constructed, resulting in a slender tail. However, ankylosaurids with average to large knobs were able to generate large impact forces.

The angular accelerations of ROM 784/UALVP 47273, UALVP 16247, and AMNH 5245/ROM 788 were similar because the proportions of the tail were all modeled from ROM 784/UALVP 47273. However, there was a great difference in impact velocities and forces because of the differences in mass and length of the tail club segment in each tail. The composite AMNH 5245/ROM 788 tail could impact with 970% more force than the ROM 784/UALVP 47273 tail.

Ankylosaurids with large knobs could deliver more forceful blows than ankylosaurids with small knobs. Impact stress results for small clubs are similar to those found for *Stegosaurus* spikes. Carpenter et al. [19] determined that a *Stegosaurus* spike could exert 360–510 N of force when swung, which they argue was more than enough to damage tissue and bone. They estimated a spike-tip impact area of 0.28 cm^2^, which would create an impact stress of 1300–1800 N/cm^2^. In contrast, ROM 784 could exert a force of 797–1127 N, using the specific tensions used by Carpenter et al. [19], and 571 N using a more realistic specific tension, creating an impact stress of 2900–5600 N/cm^2^ (29–56 MPa). Carpenter et al. [19] use ∼100 MPa (10^4^ N/cm^2^) as the maximum shear strength of living cortical bone; Currey [21] summarizes several papers which give values between 64 and 84 MPa for shear strength. The likelihood that an impacted bone would break also depends on its morphology and the way that impact stresses are transmitted through the bone (for example, a thin plate may be more likely to break than a femur). It does not appear that a *Stegosaurus* spike could puncture bone, nor could the tail club in ROM 784. This seems reasonable, as the knob in ROM 784 is small in comparison to others. UALVP 16247 represents average knob width, and could impact with a force of 962–2014 N, and exert an impact stress of 4811–10 070 N/cm^2^ (48–100 MPa). However, these results may actually underestimate impact forces and stresses in average-sized knobs, because these estimates are based on the most fragmentary specimen in this study. Average-sized knobs may have been able to break bone during impacts. An ankylosaurid with the proportions of AMNH 5245/ROM 788 could create an impact force of 7281–14 360 N, an impact stress of 36 400–71 810 N/cm^2^ (364–718 MPa), and would very likely break bone during a tail club impact. Future studies could use finite element modeling to examine tail club strikes on potential targets, such as ankylosaur ribs (for intraspecific combat) or theropod tibiae and metatarsals (for interspecific defense).

Sensitivity analyses for the functional calculations show that the bone and muscle mass, the location of the impact site, the area of impact, and the stopping time influence the impact force and stress for each tail club. Changing these parameters within biologically reasonable bounds produced increases and decreases of 5–20% for impact force and stress and indicates that the baseline results are relatively robust. Altering the muscle mass, angle of articulation, impact area, and stopping time affected the results more than altering bone mass and impact site.

Decreasing the tail club segment mass decreased the rotational inertia of the club, making it easier to wield. Reducing the mass of the tail club also increased the impact velocity, which would have allowed for a more rapid tail strike. However, there is a trade-off between reducing the mass of the tail club and increasing impact velocity, and increasing the mass of the tail club and increasing impact force and stress.

The interlocking neural spines and prezygapophyses of the handle stiffened the distal portion of the tail, providing a support for the large terminal osteoderms. The handle reduces the maximum angular acceleration of the terminal tail segment, in comparison to a tail composed of free caudal vertebrae. As such, a flexible distal tail would be able to deliver more forceful blows than a rigid tail. It may be that the handle is necessary for postural reasons, to keep the knob elevated above the ground; Coombs [1] suggested that the tail club did not drag. Or, the handle may be necessary for absorbing the shock of impact. Handles may represent a structural trade-off between maximum velocity and strength.

The haemal arch of the handle is unique among dinosaurs as a robust, nearly continuous tube of bone on the ventral side of the centra. The anterior projection has a ventral groove which receives the posterior projection of the preceding arch. This groove becomes ventrally enclosed posteriorly, and the posterior projection of the preceding arch becomes completely surrounded by the subsequent haemal arch. The haemal arch appears to be adapted to resist vertical bending of the club, and may play a role in maintaining the neutral posture of the tail (in addition to housing the caudal artery). Although in some specimens the centra are fused in the handle, in many specimens the centra are unfused, and the tube-like, robust neural arches may act as a strut which would have kept the knob held off of the ground without requiring additional muscular effort. This tube of bone would act to keep the handle from sagging, and would therefore keep the neural arches properly aligned to resist lateral bending.

In some specimens, the knob osteoderms are sharply keeled (e.g. ROM 784), whereas in others, the knob osteoderms are blunt (e.g. ROM 788). Knob osteoderms were likely covered in a keratinous sheath, which may or may not have closely matched the underlying bone in morphology. A blow from the sharp keel of an ankylosaurid knob would be more destructive than a blow from the more rounded distal end of the knob, or from the rounded faces of the knob osteoderms.

This study modeled tail club impact forces with the assumption that the lateral movement of the tail begins only at the anterior free caudal vertebra, and does not incorporate movement of the body using the hips and hindlimbs. This simplified model almost certainly underestimates the impact force of a tail club, and if ankylosaurids engaged in this behaviour then the hips and hindlimbs would probably have played an important role in tail swinging.

ROM 784 and UALVP 47273 represent smaller individuals than ROM 788 and AMNH 5245, but not proportionately to knob width [11]. ROM 784 and UALVP 47273 probably represent almost fully mature individuals. This suggests that ankylosaurid knobs were not primarily used as defensive weapons: a weapon that is not functional until very late in life would probably not have a selective advantage over a weapon that is of use earlier in life. Small juvenile *Pinacosaurus* did not have knobs at all [22]. Life history curves similar to those created for tyrannosaurids [23] would be useful in plotting growth of the knob in relation to growth of the individual, although these results may not be possible to obtain in dermal ossifications.

An alternative hypothesis is that tail clubs evolved for use in intraspecific combat, although this is difficult to test directly. Knobs may have grown only at reproductive maturity, and may have been used during courtship battles. The two competitors may have swung tail clubs at the flanks of the opponent, which can be compared with flank-butting in bovids such as *Bison bison* [24], and head-clubbing (necking) in *Giraffus camelopardalis* [25]. Flank butting in bison often results in goring and rib fractures [24], and giraffe necking can result in leg fractures, opponents being knocked unconscious, and death [25]. If ankylosaurids engaged in a similar behaviour using tail clubs, we might expect to see a larger number of rib injuries in ankylosaurids compared to other groups of dinosaurs. A survey of the occurrence of healed ribs in ankylosaurid specimens, compared to other groups of dinosaurs, could provide some indirect evidence for this possible behaviour. Tail clubs may have also been used as a display feature. Tail clubs with large knobs were undoubtedly effective deterrents against bipedal predators. However, the exclusive use of tail clubs as a defensive weapon is not supported (nor refuted) by the results of this study.

## Materials and Methods

### Osteology

This study examines tail swinging in specimens that have been referred to the most common North American ankylosaurid, *Euoplocephalus tutus*, and *Dyoplosaurus acutosquameus*, also from North America and similar in caudal morphology. Specimens of ankylosaurid pelves, caudal vertebrae, and tail clubs were photographed and measured using digital calipers and measuring tape. Each measurement was made three times and averaged. Some measurements were obtained using photographs and ImageJ [26].

### Computed tomography

Three ankylosaurid tail clubs were scanned using computed tomography (CT), to provide information on their internal structure, and to derive three dimensional models for use in volume estimates (Fig. 2). UALVP 47273 and ROM 788 have substantial portions of the handle preserved, and represent examples of small and large knobs, respectively. UALVP 16247 and does not preserve much of the handle and have average-sized knobs. UALVP 16247 and UALVP 47273 were scanned at the University of Alberta Hospital Alberta Cardiovascular and Stroke Research Centre (ABACUS), on a Siemens Somatom Sensation 64 CT scanner, at 1 mm increments. ROM 788 was scanned at CML Healthcare Imaging in Mississauga, Ontario, at 2 mm increments. All CT scans were viewed using the software programs OsiriX [27] and Mimics [28], and interpreted using a grayscale colour palette for density values. CT scans are reposited at the host institutions.

### Muscle reconstructions

In order to understand the mechanics of tail swinging, the muscles of the tail and pelvis in ankylosaurids must be reconstructed. Of particular interest are the caudal epaxial and hypaxial muscles, and some muscles of the hindlimb, in particular the *M. caudofemoralis longus* and *M. caudofemoralis brevis*. Crocodilians are used as the main comparative analogue, and are suitable for two reasons: 1) crocodilians represent one pole of the extant phylogenetic bracket for nonavian dinosaurs [29], and 2) crocodilians have long, muscular tails, which are capable of generating large forces; crocodiles use their tails actively during swimming [30], and to propel themselves into a ‘death roll’ for rotational feeding [31]. Although birds share a more recent common ancestor with ankylosaurs than do crocodilians, crocodilian tails more closely resemble those of ankylosaurs in relative length, number of vertebrae, and size of processes for muscle attachment. Muscle reconstructions in this paper use previously published studies of crocodilian anatomy [15]–[17], [32] and dinosaurian muscle reconstructions [13], [14], and comparisons with lizards and birds [18], [33] that complete the phylogenetic bracket for ankylosaurs.

### Mathematical Derivation of General Ankylosaur Tail Dynamics

Alexander et al. [34], Carpenter et al. [19], and Snively and Russell [35] have investigated the dynamics of vertebral flexion in fossil vertebrates: Alexander et al. [34] estimated tail blow energy in glyptodonts, Carpenter et al. [19] calculated impact force in *Stegosaurus* spikes, and Snively and Russell [35] investigated tyrannosaurid necks. A method similar to method 2 employed by Carpenter et al. [19] is used here, as this method is the most detailed, and using this method allows the mechanics of stegosaur and ankylosaur tail impacts to be compared Stegosaurs and ankylosaurs are both thyreophorans, yet have evolved very different putative tail weapons. Carpenter et al. [19] measured a large mounted *Stegosaurus* and modeled the tail as a series of five rigid links, with the anterior and posterior boundaries of the links defined by the large plates that occur above the vertebrae of *Stegosaurus*. Ankylosaurids were not limited by such large plates in the tail region, although many ankylosaurids (e.g. *Dyoplosaurus*) have laterally-oriented, wedge-shaped osteoderms along the lateral sides of the tail [2]. The complete caudal armour is not confidently known in *Euoplocephalus*. Therefore, osteoderms other than the knob osteoderms of the tail club are ignored, for both mass estimates and possible limits on the range of motion of the tail.

ROM 784 (*Dyoplosaurus*) has eleven free caudal vertebrae, eleven visible handle vertebrae, and a transitional free caudal vertebra was not preserved [11]. A similar number of free caudal vertebrae, including the transitional free caudal vertebra, is found in ROM 1930 (*Euoplocephalus*). No movement would have been possible between the transitional free caudal vertebra and the first handle vertebra, because the prezygapophyses of the first handle vertebra embrace the neural spine of the transitional caudal vertebra. Therefore, there would have been twelve free caudal segments and one tail club segment, for a total of thirteen segments to model in the tail. The ossified tendons are not included in this analysis, because their role in tail club swinging and impacts is unresolved.

**Figure 10:**
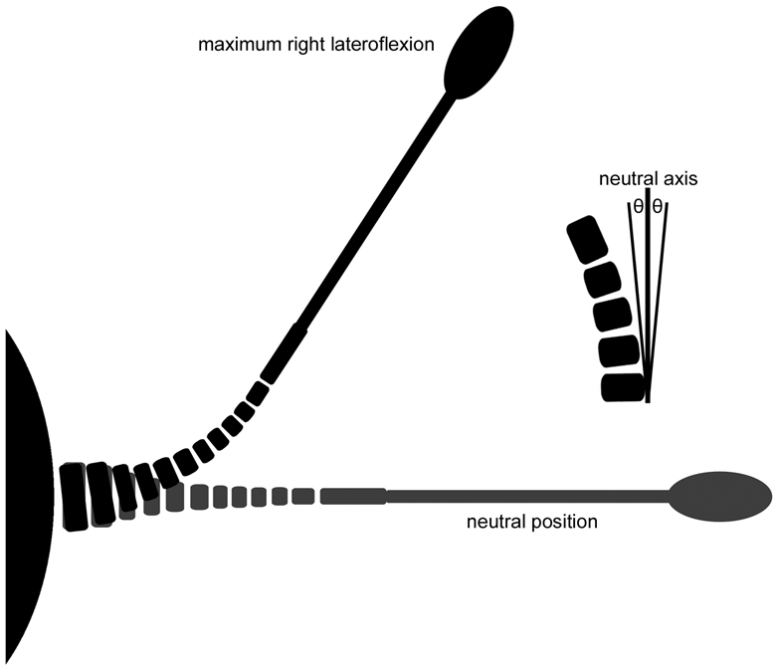
Diagram showing the approximate right lateroflexion of the tail in *Euoplocephalus*, and the definition of the half angle of articulation θ.

**Figure 11:**
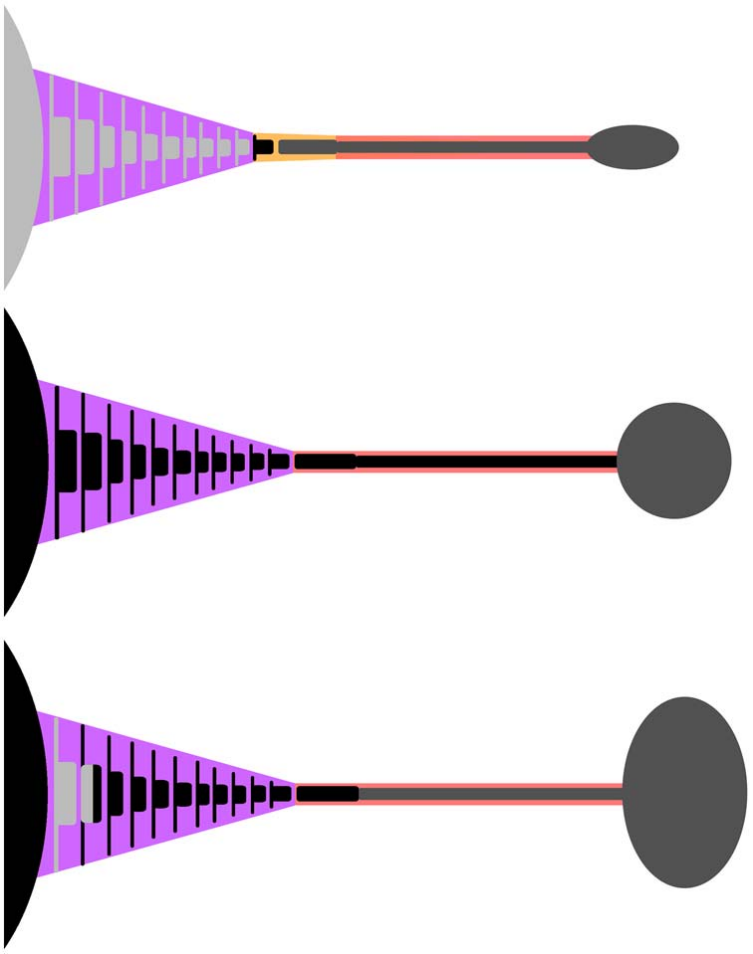
Diagrammatic representation of composite tails used in this study. A) ROM 784 (*Dyoplosaurus*)/UALVP 47273 (*Euoplocephalus*) composite tail. ROM 784 elements indicated by light grey. UALVP 47273 elements indicated by dark grey. The black vertebra represents the transitional vertebra in ROM 1930. Its presence is inferred by the gap at this location in ROM 784. The light purple area represents the free caudal tail frustum, and the dark purple area represents a single free caudal tail segment. The orange area represents the transitional tail frustum, and the pink area represents the handle volume. B) UALVP 16247 reconstructed tail. Only the knob is preserved (dark grey); the rest of the tail is reconstructed from measurements of ROM 784 (black). C) AMNH 5245/ROM 788 composite tail. AMNH 5245 elements are light grey, ROM 788 elements are dark grey, and elements reconstructed from ROM 784 are black.

The impact force of the knob is related to the acceleration and mass of the tail club segment. Actual acceleration and mass cannot be directly measured, and so must be calculated and inferred using other properties. Properties that can be directly measured on fossil specimens include length, width, and height of each vertebral segment. From these, the volume of each bone segment can be calculated, and the mass of the bone can be calculated using estimates of modern bone density. Muscle height, length, and width can be estimated for each segment, which provides approximations of muscle cross-sectional area, volume, and mass. Another property that can be directly estimated from the fossils is the angle of articulation of each segment, 2θ (where θ represents the half angle of articulation of each segment). The half angle of articulation is the maximum amount of lateral deflection from the neutral position (Fig. 10). Some specimens are incomplete or mounted in such a way that not all measurements could be taken. As such, in this study specimens of similar size are combined into composite specimens (Fig. 11). In one instance, this involves combining specimens that belong to different taxa (ROM 784, *Dyoplosaurus*, and UALVP 47273, referred to *Euoplocephalus*). The caudal vertebrae of *Dyoplosaurus* are very similar to those of *Euoplocephalus* in both size and overall morphology [11], so it is reasonable to create a composite tail for the purposes of examining general tail function in ankylosaurids.

From these properties, rotational inertia and torque can be calculated and used to calculate impact velocity, force, and stress. Carpenter et al. [19] use the following equation for rotational inertia, I:



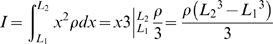
(5)


Where L_1_ is the distance from the proximal end of the segment to the base of the tail, L_2_ is the distance from the distal end of the segment to the base of the tail, ρ is the average mass density per unit length, and x is the variable of integration between L_1_ and L_2_.

Muscles pull on one half of the width of each link at the proximal end, which generates torque in each segment.
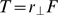


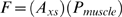



In these equations A_xs_ (in cm^2^) is the cross-sectional area of muscle at the proximal end of the segment, and P_muscle_ is the specific tension of the muscle, the force the muscle can exert per unit of cross-sectional area (N/cm^2^). A_xs_ is determined by calculating the total cross-sectional area of the tail (represented by an ellipse), and subtracting the ellipse representing the cross-sectional area of the centrum. If the rotation axis is in the centre of the segment, then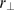 is the distance from the centre of the segment to the line of force, or the outside of the segment; this is equivalent to half of the width of the segment (w/2). Therefore:



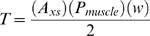
(6)


The impact velocity and impulse are related to ω, rotational velocity, and α, rotational acceleration. ω is additive along the tail, so the velocity increases from segment to segment (summation of velocities, [36]). ω and α can be related to I, T, and the angle through which each segment moves, θ.
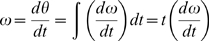


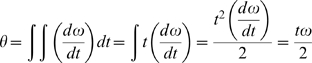



Rearranging for ω gives 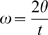.
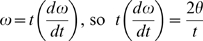


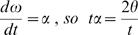



Rearranging for t gives 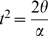


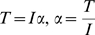


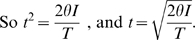



Then t can be substituted into ω = 2θ/t to express ω in terms of θ, I, and T.
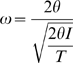


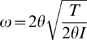


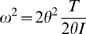


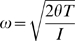



Because ω is additive along the tail segments, ω_club_ is:



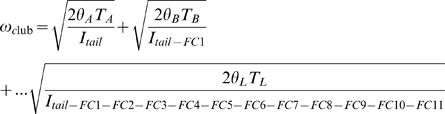
(7)


### Analysis of a small knob and tail, ROM 784/UALVP 47273

#### Determining tail volumes and masses for calculating rotational dynamics

Calculating rotational inertia and angular acceleration requires mass estimates, which are derived from estimates of bone and muscle density and volume. ROM 784 includes all free caudals (except for the final, transitional free caudal) and the entire tail club. UALVP 47273 is a partial tail club with similar proportions to ROM 784. Calculating the volume of bone and muscle in the tail requires three steps: 1) calculating the volume of the moveable, free caudal portion of the tail, 2) calculating the volume of the handle, and 3) calculating the volume of the knob. For this study, each vertebra and the subsequent disk space represent a segment (Fig. 11). Ankylosaurid vertebrae each have an approximately circular centrum in anterior view, with width exceeding height slightly in ROM 784. The neural spine and haemal spine are approximately equal in height. Centrum height and width, neural spine and haemal spine height, and transverse process length decrease posteriorly, whereas centrum length increases posteriorly.

Based on the above reconstruction of ankylosaur caudal muscles, the volume of these muscles was much greater that those of their associated neural and haemal arches. The volumes of these osseous structures are difficult to estimate and will be ignored, and only the volume of the centra will be used for calculating segmental and total muscle volume. The shape of each segment (centrum+subsequent disk space) can be represented by a truncated cone with an elliptical base (an elliptical frustum). The equation to determine the volume of any pyramidal frustum is:
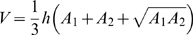



Where A_1_ is the area of the base of the pyramid, A_2_ is the area of the plane truncating the pyramid, and h is the height from A_1_ to A_2_. The area of an ellipse is:
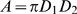



Where D_1_ and D_2_ are the major and minor axes of the ellipse. To calculate the volume of the vertebral segment, segment length l (centrum length+length of disk space), width D_1_, and height D_2_ must be known; D3 and D4 are the major and minor axes of the more posterior ellipse. Volume is then calculated as:



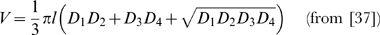
(8) (from [37])

Unfortunately, not all of these parameters are known for every segment in ROM 784. Some of the vertebrae are crushed and distorted, which yields measurements that do not necessarily decrease from one vertebra to the next. To compensate for these problems, an ‘ideal’ ROM 784 is constructed. Measurements of centrum height, width, and length were plotted as a scatterplot and slope of the lines of best fit calculated. The slope of each line is then used to calculate new heights, lengths, and widths, which are then used to calculate volume (Table 10).

**Table 10:** Actual and ideal values for dimensions of the centra in ROM 784, in mm.

Vertebral Segment #	Measured Centrum Length	Disk Space Length (approx-imate)	Total Length	Ideal Segment Length (y = −0.29x+76.171)	Centrum Height	Ideal Segment Height (y = −1.35x+73.94)	Centrum Width	Ideal Segment Width (y = −2.00x+91.36)	Volume of elliptical frustum
**1**	60.49	20	80.49	75.88	-	72.59	63.05	89.36	1514000
**2**	59.66	20	79.66	75.59	93.12	71.25	82.6	87.36	1447000
**3**	48.33	20	68.33	75.30	-	69.90	-	85.36	1381000
**4**	48.64	20	68.64	75.02	87.86	68.55	-	83.36	1317000
**5**	54.44	20	74.44	74.73	68.41	67.21	-	81.36	1255000
**6**	59.06	20	79.06	74.44	85.19	65.86	-	79.36	1194000
**7**	49.49	20	69.49	74.15	68.66	64.52	-	77.36	1135000
**8**	58.86	20	78.86	73.86	-	63.17	67.03	75.36	1078000
**9**	-	-	-	73.57	-	61.82	52.64	73.36	1022000
**10**	-	-	-	73.28	-	60.48	-	71.36	9682000
**11**	-	-	-	72.99	74.91	59.13	62.64	69.36	9159000
				72.70		57.79		67.36	

Calculating the volume of muscle in the flexible portion of the tail is more complicated. In crocodilians, the musculature of the tail is to a certain extent limited by the vertebrae, but the cross-sectional profile of the tail changes greatly from anterior to posterior [15]. A conservative reconstruction of the muscles of the tail of ankylosaurids would have an elliptical cross-sectional outline, with none of the muscles bulging past the transverse processes or neural and haemal spines. If this is the case, the shape of the tail as a whole would mimic the shape of the centra, and the tail can be modeled as a series of truncated elliptical cones just like the centra. This reconstruction ignores the muscles of the pelvis that continue caudally.

The heights of the neural and haemal spines, and lengths of the transverse processes, were measured in ROM 784. Neural spine height was measured from the bottom of the neural canal to the distal tip of the spine, perpendicular to the anteroposterior axis of the centrum. Haemal spine height was similarly measured from the top of the haemal canal to the distal tip of the spine. As before, measurements for all elements could not be obtained as some vertebrae were missing some or all of these elements. The ‘ideal’ neural spine, haemal spine, and transverse process values were calculated as above. The height of a tail segment is the sum of the heights of the haemal spine, centrum, and neural spine. The width of a tail segment is the sum of the width of the centrum and the length of both transverse processes. The volumes were calculated as for the centra (Table 11). To obtain the volume of the muscles, the volume of the centra is subtracted from the total volume of the tail.

**Table 11:** Actual and ideal values for dimensions of the tail in ROM 784, in mm.

Vertebral Segment #	Measured neural spine height	Ideal neural spine height (y = −4.22x+115.19)	Ideal haemal spine height (y = −4.22x+115.19)	Measured transverse process length	Ideal transverse process length (y = −10.46x+113.36)	Ideal total height (neural spine+centrum height+haemal spine)	Ideal total width (centrum width+(2* transverse process))	Volume of elliptical frustum (mm^3^)
**1**	104.91	110.97	110.97	130.77	102.90	294.53	295.16	19580000
**2**	99.01	106.75	106.75	111.36	92.44	284.75	272.24	17330000
**3**	94.62	102.53	102.53	57.54	81.98	274.96	249.32	15190000
**4**	106.65	98.31	98.31	43.11	71.52	265.18	226.40	13180000
**5**	108.36	94.09	94.09	54.91	61.06	255.39	203.48	11290000
**6**	101.57	89.87	89.87	41.05	50.6	245.61	180.56	9515000
**7**	88.5	85.65	85.65	31.98	40.14	235.82	157.64	7859000
**8**	84.29	81.43	81.43	32.41	29.68	226.04	134.72	6321000
**9**	66.81	77.21	77.21	46.38	19.22	216.25	111.80	4897000
**10**	64.91	72.99	72.99	0	8.76	206.47	88.88	3667000
**11**	68.96	68.77	68.77	0	0	196.68	69.35	3004000
-	-	64.55	64.55	-	0	186.89	67.35	-

The handle vertebrae are not as easily represented geometrically, and measurements of the heights, widths, and lengths of the centra were not possible in ROM 784 because the specimen is partially embedded in matrix. However, a partial tail club with similar vertebra and knob proportions (UALVP 47273) has been CT scanned. This specimen can be scaled to the size of ROM 784, and measurements of the volume of this specimen can be substituted for ROM 784.

Volume is estimated using ImageJ to trace areas of interest in CT slices, then multiply by slice thickness [38]. CT scan data was imported in OsiriX, and then individual slices were exported as TIFF files at 10 mm intervals (plus an additional slice representing 5 mm), totaling a length of 475 mm. These images were analyzed using ImageJ. Regions of interest (ROIs) were traced manually based on density contrasts in the image. ROIs for the handle vertebrae included the total cross-sectional areas, and the areas of the neural arch plus neural canal, neural canal, centrum, haemal arch, and haemal canal. The total cross-sectional area is multiplied by slice thickness to find the volume of each slice, and these results are then summed to find the volume of the club. Volumes of the compact neural and haemal arches, cancellous centra, and ‘empty’ neural and haemal canals can be calculated in the same manner. Using this method, the total volume of the handle vertebrae in UALVP 47273 is 1025 cm^3^.

**Figure 12:**
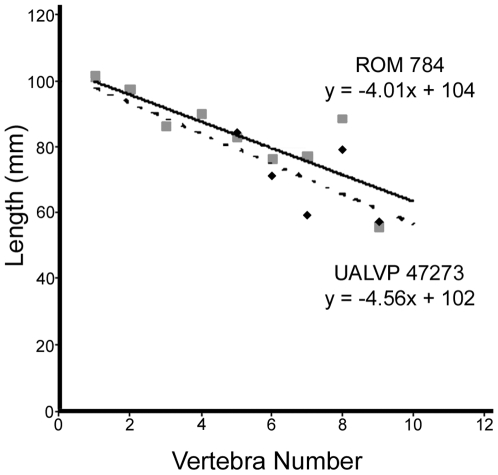
Graph comparing the length of the neural spine of the handle vertebrae in ROM 784 and UALVP 47273. ROM 784 is represented by the solid line and squares. UALVP 47273 is represented by the dashed line and diamonds. Source data are in Table 12.

ROM 784 is slightly larger than UALVP 47273. The proportions of the knob cannot be used to scale UALVP 47273 to ROM 784, because knob size does not seem to be correlated with vertebra size [11]. Measurements of the length of the neural spine on each handle vertebra were plotted on a scatterplot, and the slope was calculated using a linear regression. The slope was similar for both ROM 784 (−4.01) and UALVP 47273 (−4.56), and so the length of the neural spine was chosen as an appropriate scaling measure (Fig. 12). ROM 784 is 109% the length of UALVP 47273 using this measure (Table 12). The width of the knob of ROM 784 is 107% that of UALVP 47273.

**Table 12:** Comparison of handle vertebra neural spine length, and knob width (in mm), in ROM 784 and UALVP 47273.

	Handle Vertebra neural spine length									Avgas	Knob width
	1	2	3	4	5	6	7	8	9		
**ROM 784**	101.14	97.27	86.07	89.61	82.65	76.26	77.08	88.31	55.52	83.77	166
**UALVP 47273**	-	-	-	-	84.16	70.98	59.4	79.11	57.3	70.19	155
**UALVP 47273 as a % of ROM 784**	-	-	-	-	98.21	107.44	129.76	111.63	96.89	108.79	107.10
**ROM 784 as a % of UALVP 47273**	-	-	-	-	101.83	93.08	77.06	89.58	103.21	92.95	93.37

The length of the club of ROM 784, from the anterior of the first handle vertebra to the posterior terminus of the knob, is 127 cm. The measured length of the handle, plus the length of the knob, in UALVP 47273 is 71 cm. Scaling by 1.09 gives a length of 77 cm. Scaling the measured volume by 1.09^3^ gives a volume of 1330 cm^3^. UALVP 47273 is an incomplete club; subtracting 77 cm from a total length of 127 cm gives a missing length of 50 cm. The average cross-sectional area of each slice is 21 cm^2^, which scaled to ROM 784 is 25 cm^2^. Multiplying this average by 50 cm provides an estimate of 1260 cm^3^ for the volume of the missing area in UALVP 47273. This actually underestimates the likely missing volume, because in ROM 784 the centra of the first two handle vertebrae are slightly larger than the rest of the centra. Summing the scaled up volume of the measured portion of UALVP 47273 (1220 cm^2^), and the estimated volume of the missing portion (1260 cm^3^), yields a bone volume of 2470 cm^3^. ROM 784 is probably missing a vertebra in the middle of the series [11]. To model this vertebra, an additional, twelfth ‘ideal’ vertebra was constructed using the ‘ideal’ free caudal equations, and the volume was calculated as 865 cm^3^.

Calculating the total volume of the handle, with muscles reconstructed, is more difficult than with the free caudals. There are fewer osteological correlates for muscle attachments in the handle vertebrae. As discussed previously, the width between the medial sides of the two major knob osteoderms in dorsal view must have been the maximum width of the handle muscles. A CT scan cross-sectional slice of the handle of UALVP 47273 provided the basis for reconstructing the musculature. This reconstruction was then measured using ImageJ, giving a cross-sectional area of 60 cm^2^ (71 cm^2^ scaled to ROM 784).

The first two handle vertebrae in ROM 784 are larger than the more posterior handle vertebrae, and have small bumps where the transverse processes are located in the more anterior caudals. To approximate the musculature of the free caudals tapering onto the handle, a frustum from the anterior of the transitional free caudal vertebra to the posterior of the second handle vertebra was calculated. The length of the first two handle vertebrae in ROM 784 is 19 cm. Using this length, the ‘ideal’ dimensions for the transitional free caudal musculature, and an area of 71 cm^2^ as the top of the frustum, a volume of 7640 cm^2^ was calculated. The remaining length of the club is 108 cm. Subtracting the length of the knob (23 cm in UALVP 47273, scaled to 25 cm) gives the remaining length of handle for which total volume must be calculated. The handle vertebrae do not taper much posteriorly, and for the purposes of this study it is assumed that the total volume of the tail in the handle did not taper posteriorly either. Therefore, the cross-sectional area of 71 cm^2^ can be multiplied by the length to obtain a volume of 4970 cm^2^.

With the total volumes of the various tail segments, and the volumes of the vertebrae, the volume of muscle can be calculated. The total volume of the tail (excluding the knob) is 12610 cm^3^, and the total volume of the vertebrae is 3250 cm^3^. Subtracting the volume of the vertebrae from the total volume gives a muscle volume of 9360 cm^3^.

The knob of ROM 784 is not easily modeled using simple geometry, and measurements of all dimensions could not be obtained because the knob is partially embedded in matrix. Instead, the volume of UALVP 47273 was calculated by tracing the area of CT scan slices in ImageJ and multiplying by slice thickness (1 mm). Traced areas included the total area of the knob, and cancellous area of each osteoderm. The volume of the knob of UALVP 47273 is 1550 cm^3^, and the scaled volume is 2010 cm^3^.

It is difficult to reconstruct with certainty the size and shape of the probable keratinous sheath that would have covered each of the knob osteoderms. In many horned ungulates, the morphology of the horny sheath does not closely match the size and shape of the inner bony horn core [39]. Keratinous coverings in *Alligator mississippiensis* osteoderms appear to conform more closely to the shape of the underlying osteoderm, and particularly augment the shape of the keel, if present [40]. A specimen of the basal thyreophoran *Scelidosaurus* with preserved integument indicates that thyreophoran osteoderms were covered in a thin layer of skin or horny keratin [41]. The size of the keratinous sheath probably does not greatly affect the rotational inertia of the tail, and is not included in the following calculations. However, the size and shape of the sheath would play a role in absorbing stress and strain upon impact: Snively and Cox [20] found that the thickness of keratin covering a pachycephalosaur dome reduced the strain in the bone during impacts.

#### Determining the angle of articulation between free caudal vertebrae: range of motion and angular deflections of the tail

An important variable for determining forces, velocities, and impulses is the amount of rotation possible between each free caudal vertebra. Based on manual manipulation of articulated ankylosaurid vertebrae (ROM 1930), free caudals appear to have had limited vertical motion, but were capable of lateral motion. For the purposes of this study, it is assumed that tail club strikes occurred through lateral movement of the tail. The maximum angle of rotation is the maximum left and right divergence from midline. The maximum half angle of rotation is the maximum divergence in one direction from the midline. Ideally, a complete specimen with all or most vertebrae preserved and prepared out of the matrix could be manipulated to manually measure the maximum half angle of rotation between each vertebra. Whereas ROM 784 preserves almost all of the caudal vertebrae, it is embedded partially in matrix and the vertebrae cannot be moved to measure angles. Several other specimens have two or three vertebrae in sequence and prepared out of the matrix (ROM 1930, AMNH 5404), but in these specimens the zygapophyses are not complete between vertebrae, and so the maximum half angle of rotation could not be determined. An alternative method for determining the half angle of rotation is presented here.

Stevens and Parrish [42] found that the synovial capsules of the pre- and postzygapophyses or extant birds constrained the amount of movement between each vertebral joint. Zygapophyseal facets must overlap by approximately 50%. Dzemski and Christian [43] examined flexibility in ostrich (*Struthio camelus*) necks and skeletonized necks of camels (*Camelus bactrianus*) and giraffes (*Giraffus camelopardalis*), and found that maximum lateral flexion is limited by the overlap between the zygapophyseal joint facets. In lateral flexion of ostrich necks, the overlap of the joint facets was equivalent to the rim of one facet covering between one eighth and one quarter the long diameter of the corresponding facet. Muscles along the neck reduced the lateral flexion if a long segment of the neck was flexed. Extreme lateral flexions of the necks of living ostriches were close to the values obtained from neck skeletons. Dzemski and Christian [43] found that the maximum intervertebral lateral flexions in the necks of the ostrich and camel, which both have very flexible necks, are below 25°.

**Figure 13:**
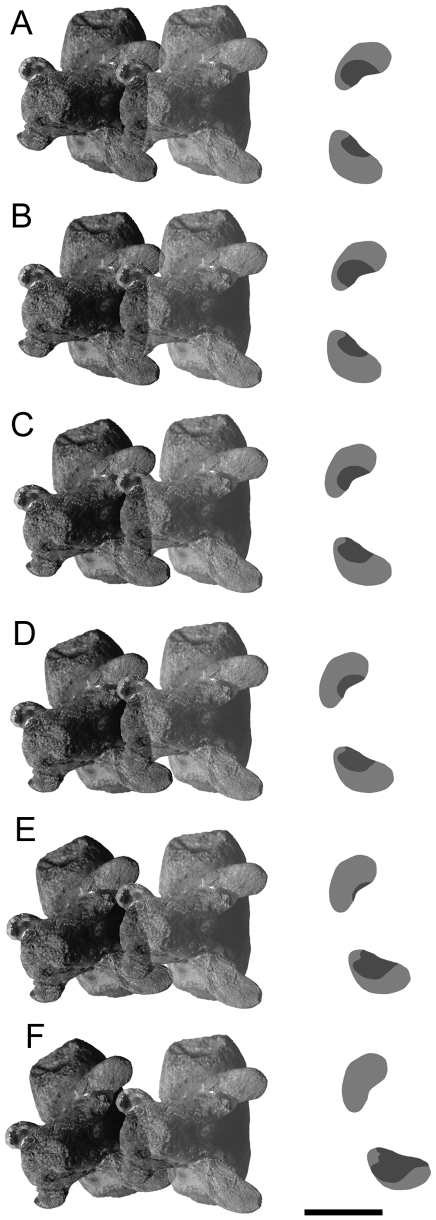
Determining the maximum angle of rotation in ankylosaurid free caudal vertebrae. A dorsal view of TMP 2007.20.80 is on the left, and a 3% larger copy is on the right. The vertebrae are separated by a 2 cm gap representing the intervertebral cartilage. The left vertebra is rotated from 0 to 25 degrees, in 5 degree increments, from A to F. The articular faces of the prezygapophyses in light grey, and the area covered by the postzygapophyses in darker grey, are shown for each rotation. Scale bar equals 5 cm.

The studies by Stevens and Parrish [42] and Dzemski and Christian [43] provide a guideline by which maximum angles of rotation can be determined in ankylosaur tails: the amount of contact between the zygapophyseal joints. TMP 2007.20.80 (*Euoplocephalus*), an isolated free caudal vertebra, has complete prezygapophyses and postzygapophyses. In ROM 784 (*Dyoplosaurus*) and ROM 1930 (*Euoplocephalus*), each successive free caudal vertebra is approximately 3% smaller in width than the preceding vertebra. A dorsal photograph of TMP 2007.20.80 was rotated by 0°, 5°, 10°, 15°, 20°, and 25°. The axis of rotation follows that of Snively and Russell [35], at approximately the midpoint between the prezygapophyses. The photograph was scaled by 103% to create a preceding vertebra. The rotated original photograph and enlarged photograph were overlain so that the zygapophyses articulated (Fig. 13). The prezygapophyses of the rotated image and the area covered by the postzygapophyses of the enlarged image were measured in ImageJ (Table 13). The original photograph is not perfectly aligned, so that the non-rotated original photo and enlarged photo do not articulate perfectly. This explains why the area in contact in the right zygapophyses is less than 25% when the angle of rotation is zero. However, this method provides an effective way to estimate the maximum angle, even if the photograph is not perfectly aligned, or if the specimen is slightly taphonomically distorted. The maximum lateral flexion of the caudal vertebrae are estimated to have been between 5° to 10° from the neutral position, and would have almost certainly been less than 20°.

**Table 13:** Area of overlap between successive zygapophyses, in mm.

Angle	Area of left prezyg-apophysis	Area of right prezyg-apophysis	Area of left postzyg-apophysis	Area of right postzyg-apophysis	Left postzyg-apophysis area/left prezygapophysis area, %	Right postzyg-apophysis area/right prezygapophysis area, %
**0**	7.12	7.84	2.62	1.39	36.8	17.7
**5**	7.12	7.84	2.65	1.63	37.2	20.8
**10**	7.12	7.84	1.85	2.24	25.9	28.6
**15**	7.12	7.84	1.34	2.34	18.8	29.8
**20**	7.12	7.84	0.42	3.15	5.89	40.18
**25**	7.12	7.84	0	4.67	0	59.54

#### Calculating *T, I,* and *ω*

Table 1 summarizes the volume of bone and muscle, proximal cross-sectional area of muscle, mass of bone and muscle, total mass, length, and total mass per unit length for each segment of the tail.

Rotational inertia (Table 2) for each segment was calculated using Equation 5, 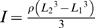, where ρ is the mass per unit length, calculated in Table 6. L_2_ and L_1_ change for each segment and each I.

Torque (Table 3) is calculated for each segment using Equation 6, 
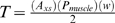
. Carpenter et al. [19] used 39 N/cm^2^ and 78 N/cm^2^ as the upper and lower bounds for the range of forces that muscles can exert. Snively and Russell [35] note that the amount of force a muscle can exert is related to its cross-sectional area and length, the geometry of muscle fibers, and the composition of muscle fibers, and that muscle velocity is related to fibre type and operating temperature. The type of fibers associated with ankylosaurid tail muscles is difficult to assess, however, Snively and Russell [35] note that the superficial neck muscles of many archosaurs appear to have fibres that can contract rapidly, and so it may be possible that ankylosaur tails had a similar fibre type. The body temperature of ankylosaurids is unknown: Seebacher [44] suggested that ankylosaurs did not evolve endothermy, whereas Gillooly et al. [45] provided evidence that most large dinosaurs were inertial homeotherms. For the purposes of this study, it is assumed that ankylosaurids had muscle physiology comparable to those of extant homeotherms.

Muscle volume, fiber length, and pennation angle are used to estimate the physiological cross-sectional area (PCSA) of a muscle, which can be related to muscle force. The force a muscle produces per unit area is its specific tension (ST), and specific tension multiplied by the PCSA yields the contraction force of the muscle [46]. It is impossible to estimate most of the factors involved in calculating PCSA for fossil taxa, but PCSA is probably close to anatomical cross-sectional area (ACSA) in fusiform muscles [35]. Specific tension can be estimated from studies of extant vertebrates, as it is relatively uniform in vertebrate muscle that is shortening by concentric contraction [35]. Specific tension has been found to range between 15 to 24 N/cm^2^ in a variety of extant vertebrates [47]–[50]. Ankylosaurid muscle forces are calculated using 20 N/cm^2^ as a typical specific tension for concentric contraction. To facilitate comparisons with Carpenter et al.'s [19] results for *Stegosaurus*, forces are also calculated using their values for specific tension (39 N/cm^2^ and 78 N/cm^2^).

The ω term is calculated using 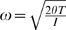. The sum of the ω terms (Table 4) is Equation 7,
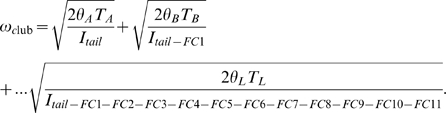



Ankylosaurids may have initiated a tail swing from the neutral position of the tail extended straight from the hips and without any lateroflexion between the caudal vertebrae. However, a more forceful impact would be achieved if the tail was swung from the maximum deflection of one side to the maximum deflection on the other side. Using 7.5° as an average half angle of articulation, the angle of articulation between each free caudal vertebra was 15°.

#### Sensitivity Analyses for the ROM 784/UALVP 47273 club

There are several factors that could affect the results in ROM 784/UALVP 47273 that should be examined:

1. Bone mass. Differences in the density of cancellous and compact bone can affect the mass estimates for each segment, and in particular the mass of the tail club segment.
2. Muscle reconstructions. Differences in the amount of muscle reconstructed can affect the mass of each segment and the cross-sectional area used to calculate torque.
3. Angle of articulation between free caudal vertebrae. The angle of articulation is difficult to determine precisely, and may be too low or too high. The maximum angle of articulation may also change posteriorly along the tail.
4. Site of impact on club. The site of impact could be more posterior or anterior on the knob.
5. Area of impact. The area of impact could be greater or smaller, depending on the shape of the keratinous sheath, and whether the impact is along a sharp or blunt keel, or on the rounded surfaces of the knob osteoderms.
6. Stopping time.

Each of these variables was changed systematically with the composite ROM 784/UALVP 47273 tail. Carpenter et al. [19] use 1.98 g/cm^3^ when estimating segment mass. Ankylosaurid handle vertebrae have cancellous centra and compact neural and haemal arches, and the knob is predominantly cancellous. To understand the role that bone mass plays in tail impact forces, a more accurate estimate of mass is needed. In the baseline analysis, the neural arch, haemal arch, and transverse processes were not modeled, and they are again excluded here. Additionally, changes in mass affect the calculations for rotational inertia and impulse. Because the tail club segment is so much larger than the rest of the tail segments, and because only the tail club segment is used to calculate impulse, it is reasonable to exclude the free caudal vertebrae from this sensitivity analysis.

The relative proportions of compact vs. cancellous bone in the handle vertebrae was determined by using ImageJ to calculate the cross-sectional area of the centrum and the neural and haemal arches in several transverse sections of the handle. The centrum was approximately 38% the total cross-sectional area of a handle vertebra. Extrapolating this to the handle as a whole (including the transitional vertebra), the volume of cancellous centra was 1251.10 cm^3^, and the volume of compact neural and haemal arches was 2090 cm^3^. Using average density values for cancellous (1 g/cm^3^) and compact bone (2 g/cm^3^), also used by Snively and Cox [20], yields a mass of 1250 g and 4170 g, respectively. The knob is varying densities of cancellous bone with a relatively thin layer of compact bone, and is here modeled as cancellous bone (1 g/cm^3^), giving a mass of 2010 g. The bone mass of the tail club segment is therefore 7430 g, which is less than the estimate using 1.98 g/cm^3^ as an average. The total mass of the tail club segment (including muscles) is 16.79 kg.

The amount of muscle that would have powered the tail is subjective. For the baseline analysis, it was assumed that muscles did not bulge outwards past the neural and haemal spines and the transverse processes. Reconstructing the muscles in this way allows the tail to be modeled as a larger frustum containing the centra frustum. However, the muscles may have been much larger than depicted in this reconstruction. The areas of two reconstructions (Fig. 9) were compared in ImageJ. TMP 85.26.70 was reconstructed with conservative musculature, and with bulging muscles. The cross-sectional area of the segment was 1310 cm^2^ for the conservative estimate, and 1870 cm^2^ for the larger estimate. The larger reconstruction is 143% the size of the conservative estimate. Using this value, the values of the cross-sectional areas of the tail segments in the baseline analysis can be scaled upwards, and the maximum force recalculated. The half width of each segment is left unchanged, because the reconstructed muscles do not necessarily bulge laterally past the transverse processes.

In the baseline analysis, 15° was selected as a probable maximum angle of articulation between each pair of the free caudal vertebrae. Maximum angles of articulation of 5°, 10°, and 20°, and the effects of decreasing the amount of rotation posteriorly along the tail were examined, starting at 15° and moving to 0° at the articulation between the intermediate caudal and first handle vertebra. The degree of rotation between the free caudal vertebrae was calculated by graphing the rotation between the pelvis and first free caudal as 15° and the rotation between the intermediate caudal and first handle vertebra as zero, then taking the slope of the line (y = 1.25x+16.25) and calculating the amount of rotation for the vertebrae in between. The variables r, m_club_, t_stop_, A_impact_, and I_club_ are the same as those used in the baseline analysis.

If the vertebrae of the handle were not fused and rigid, and instead were able to rotate freely like the free caudal vertebrae, then impact velocity in the knob would increase, as would impulse, force, and stress. Following the same procedure as for the baseline analysis, with the main changes being the calculation of torque and rotational inertia for the extra segments, the value of ω_club_ is determined to be 8.31 rad/s to 11.76 rad/s. To examine the role of the handle in tail swinging, a hypothetical tail composed entirely of free caudal vertebrae is constructed. In ROM 784, there are at least eleven, and probably twelve vertebrae in the handle. With eleven free caudal vertebrae and one missing transitional vertebra, the total number of vertebrae would have been 24. Even assuming that all of the vertebrae were free caudal vertebrae, the knob would still enclose the last two caudal vertebrae. This means there would be 23 segments (22 vertebrae and the knob). The length of the knob is 23 cm, which scaled to ROM 784 is 25 cm.

Changing the site of impact on the club changes the value of r, the distance from the base of the tail to the site of impact. For this analysis, the club impact points are assumed to be near the distal tip of the club (10 cm from the distal terminus, r = 2.06 m) and near the anterior margin of the knob (29 cm from the distal terminus, r = 1.87).

The area of impact is determined by the shape of the keratinous sheath that would have covered the knob. Because the shape of the sheath is unknown, the area of impact is speculative. The area of impact may have varied greatly depending on where the site of impact was, and what the knob was impacting. In the baseline study, an area of 0.20 cm^2^ was chosen as a reasonable approximation. The bluntness or sharpness of the keel of the sheath would also affect the impact area.

### Analysis of a large tail and knob, AMNH 5245/ROM 788

Whereas ROM 784 and UALVP 47273 are two of the smallest tail clubs, ROM 788 and AMNH 5245 (both *Euoplocephalus*) have the widest knobs encountered during the course of this research. A complete caudal series is not available in either of these specimens. ROM 788 includes the knob and most of the handle. AMNH 5245 includes the knob, some of the handle, and two anterior free caudals which may represent the first and second free caudals. An ideal free caudal series can be constructed in the same manner as for ROM 784. Measurements of the vertebra were only possible in the first free caudal, so for the purposes of these estimates the proportions of the vertebrae in AMNH 5245 are assumed to decrease in the same manner as ROM 784. This is a reasonable assumption because the proportions of ROM 1930 (which has overall larger vertebrae than ROM 784) decrease at the same rate as in ROM 784. The width of the centrum in the first vertebra could not be calculated because it is broken. To estimate the width, the width:height ratio for each ROM 784 free caudal segment (vertebra and disk space) was determined, then used to calculate the width in AMNH 5245. To calculate the proportions of ideal AMNH 5245, the same slope value is used as that calculated for ideal ROM 784, and the intercept value is changed to the measurement of the first free caudal vertebra in AMNH 5245. It is also assumed that AMNH 5245 has 11 free caudal vertebrae and one transitional vertebra, as in ROM 784. The musculature of the tail in AMNH 5245 is modeled in the same manner as for ROM 784.

The handle vertebrae of AMNH 5245 are partially embedded in matrix, but a tail club with similar vertebra and knob proportions (ROM 788) is available. In this case, AMNH 5245 is the less complete specimen, so it is scaled to the size of ROM 788. ROM 788 was CT scanned and the data analyzed in ImageJ as for UALVP 47273.

AMNH 5245 is scaled to the size of ROM 788 using the length of the neural spine. Measurements of the length were plotted on a scatterplot and the slope was found to be similar in ROM 788 (4.275) and AMNH 5245 (3.426). ROM 788 is 158% the length of AMNH 5245. The width of the knob of AMNH 5245 is 103% the width of ROM 788. As such, all of the free caudal vertebrae segments are scaled 158%.

The length of the preserved part of the club in ROM 788 is 126 cm, and eight vertebrae are visible. Ten vertebrae are visible in ROM 784. If ROM 788 had the same number of vertebra in the tail, and the average length of the vertebra is 9 cm (the length of the club minus the length of the knob, 75 cm, divided by 8 vertebrae), then the length of the tail club (handle+knob+missing vertebrae) would have been 147 cm. Included in the tail club segment for modeling purposes is the transitional vertebra, with an estimated length of 9 cm (scaled to 14 cm), giving a total tail club segment length of 161 cm.

The average cross-sectional area of each handle CT slice is 45 cm^2^, which multiplied by the length of the handle (94.58 cm) gives a bone volume of 4265 cm^2^. The transitional vertebra has a volume of 12120 cm^2^ when scaled to ROM 788. The volume of the knob was partially measured using ImageJ as for UALVP 47273; however, the knob was wider than the field of view of the scanner and the lateral edges of the knob osteoderms were not scanned. The missing portion of the knob osteoderms can be represented by an ellipsoid, where the volume is:



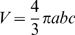
(9)


Where a, b, and c are the three axes of the ellipsoid. The axes a and b (length and width) were measured by overlying a semi-transparent coronal CT section of ROM 788 over a dorsal photograph of the specimen, and measuring the length and width of the missing part of each osteoderm in ImageJ. The height (c) of the missing portion of each osteoderm was measured from a transverse CT section in ImageJ. The measured volume of the knob was 20810 cm^3^, and the missing volume of the left and right osteoderms was 14120 cm^3^ and 18080 cm^3^, respectively. This gives a total volume of the knob of 53000 cm^3^. The bone volume of the tail club segment (transitional free caudal vertebra+handle vertebrae+knob) totals 69390 cm^3^.

The muscle volume is calculated by determining the total volume of the tail club and subtracting the volume of the transitional free caudal and handle vertebrae. The maximum width of the handle muscles is the width between the major osteoderms of the knob, which for ROM 788 was measured as 19 cm in ImageJ. In UALVP 47273, the average cross-sectional area of the reconstructed tail (muscles+vertebrae) was 60 cm^2^, and the width between the osteoderms was 10 cm. Assuming that the muscles in ROM 788 are proportionately larger, the average cross-sectional area of the tail is 117 cm^2^. Multiplying this by the length of the handle (95 cm) gives a tail volume of 11100 cm^3^, and subtracting the volume of the handle vertebrae gives a muscle volume of 6790 cm^3^. The transitional tail segment is 14500 cm^3^ (scaled to ROM 788), and subtracting the volume of the vertebra gives a muscle volume of 10780 cm^3^. Using these volumes, torque, rotational inertia, and angular acceleration are calculated in the same manner as for ROM 784/UALVP 47273.

### Dynamics of a mid-sized tail and knob, UALVP 16247

ROM 784/UALVP 47273 and AMNH 5245/ROM 788 represent the extreme ends of the range of widths in knobs measured in this study. Most tail clubs are around 40 cm wide, and two examples of average-sized tail clubs were CT scanned (UALVP 16247 and TMP 83.36.120). These are both fragmentary clubs with only the knob preserved in UALVP 16247 and a small fragment of the handle in TMP 83.36.120. As such, any estimates of lengths, volumes and masses will be more tentative for these clubs compared to ROM 784/UALVP 47273 and AMNH 5245/ROM 788. Nevertheless, an estimate can be made by ‘extruding’ the handle from the knob, and then reconstructing the free caudal vertebrae posteriorly to anteriorly using information from the ‘ideal’ ROM 784 vertebrae.

#### Estimates of bone and muscle mass and volume for UALVP 16247

UALVP 16247 represents an average-sized tail club knob. It is the most fragmentary specimen in this study, as it is an isolated knob lacking a handle. However, a rough estimate of tail dimensions can be made for UALVP 16247, to provide estimates of impact forces for the most common knob size.

A CT scan of UALVP 16247 was measured as for UALVP 47273 and ROM 788. The volume of the knob was determined to be 6486 cm^3^. Some of the terminal handle vertebrae are visible in transverse section within the knob. Several cross-sectional areas were traced and averaged to provide an estimate for the average cross-sectional area of the handle, which can be compared to the values obtained for UALVP 47273 and ROM 788 (Table 14).

**Table 14:** Cross-sectional area of UALVP 16247 handle and comparisons with ROM 788 and UALVP 47273.

Specimen	Average cross-sectional area of the handle	UALVP 16247 as a % of other specimens
**ROM 788**	45.10	80.90%
**UALVP 47273**	21.15	172.5%
**UALVP 47273 scaled to ROM 784**	25.25	144.5%
**UALVP 16247**	36.48	-

ROM 784/UALVP 47273 represents a more complete composite specimen than AMNH 5245/ROM 788. The value of UALVP 47273 scaled to ROM 784 (144.5%) is thus used as the scaling factor for UALVP 16247. This can be used to scale the proportions of the free caudal vertebrae, handle vertebrae, and muscles in ROM 784/UALVP 47273 to reconstruct the missing elements in UALVP 16247. The square root of 144.5% is used to determine the linear proportions of the vertebrae, using the ideal values of ROM 784, as well as the proportions of the tail segments.

The volume of the tail club can be calculated using proportions from UALVP 47273 scaled to ROM 784. The length of the ROM 784 tail club is 127 cm, and the length of the handle (total club length minus the length of the knob) is 101 cm. The length of the handle in UALVP 16247 is therefore 121 cm, and multiplying by the average handle cross-sectional area gives a handle vertebrae volume of 4430 cm^3^. The estimated cross-sectional area of the handle tail segment in ROM 784/UALVP 47273 was 72 cm^2^, which scaled to UALVP 16247 is 103 cm^2^. The volume of the handle tail segment is therefore 12500 cm^3^. The same method for calculating torque and rotational inertia is employed here as for ROM 784/UALVP 47273 and AMNH 5245/ROM 788. Tables including the mass, torque, and rotational inertia are not included.
